# Activating and Attenuating the Amicoumacin Antibiotics

**DOI:** 10.3390/molecules21070824

**Published:** 2016-06-24

**Authors:** Hyun Bong Park, Corey E. Perez, Elena Kim Perry, Jason M. Crawford

**Affiliations:** 1 Department of Chemistry, Yale University, New Haven, CT 06520, USA; hyunbong.park@yale.edu (H.B.P.); corey.perez@yale.edu (C.E.P.); 2 Chemical Biology Institute, Yale University, West Haven, CT 06516, USA; e.kim.perry@gmail.com; 3 Department of Ecology & Evolutionary Biology, Yale University, New Haven, CT 06520, USA; 4 Department of Microbial Pathogenesis, Yale School of Medicine, New Haven, CT 06536, USA

**Keywords:** natural product, insect pathogen, isocoumarin, biosynthesis, genome mining, orphan pathway

## Abstract

The amicoumacins belong to a class of dihydroisocoumarin natural products and display antibacterial, antifungal, anticancer, and anti-inflammatory activities. Amicoumacins are the pro-drug activation products of a bacterial nonribosomal peptide-polyketide hybrid biosynthetic pathway and have been isolated from Gram-positive *Bacillus* and *Nocardia* species. Here, we report the stimulation of a “cryptic” amicoumacin pathway in the entomopathogenic Gram-negative bacterium *Xenorhabdus bovienii*, a strain not previously known to produce amicoumacins. *X. bovienii* participates in a multi-lateral symbiosis where it is pathogenic to insects and mutualistic to its *Steinernema* nematode host. Waxmoth larvae are common prey of the *X. bovienii*-*Steinernema* pair. Employing a medium designed to mimic the amino acid content of the waxmoth circulatory fluid led to the detection and characterization of amicoumacins in *X. bovienii*. The chemical structures of the amicoumacins were supported by 2D-NMR, HR-ESI-QTOF-MS, tandem MS, and polarimeter spectral data. A comparative gene cluster analysis of the identified *X. bovienii* amicoumacin pathway to that of the *Bacillus subtilis* amicoumacin pathway and the structurally-related *Xenorhabdus nematophila* xenocoumacin pathway is presented. The *X. bovienii* pathway encodes an acetyltransferase not found in the other reported pathways, which leads to a series of *N*-acetyl-amicoumacins that lack antibacterial activity. *N*-acetylation of amicoumacin was validated through in vitro protein biochemical studies, and the impact of *N*-acylation on amicoumacin’s mode of action was examined through ribosomal structural analyses.

## 1. Introduction

The amicoumacins have been identified in select Gram-positive *Bacillus* and *Nocardia* species and belong to a larger group of bacterial dihydroisocoumarin natural products [1,2]. The structure of amicoumacin A from *Bacillus pumilus* and its anti-inflammatory and antiulcer activities were first reported in 1981–82 by Itoh and colleagues [3,4]. A number of amicoumacin analogs have since been structurally and functionally characterized [5,6,7,8,9] and synthetic routes have been developed to access their core scaffolds [10,11]. These secondary metabolites also harbor potent antibacterial activities against clinically-relevant bacterial pathogens, such as *Helicobacter pylori* and methicillin-resistant *Staphylococcus aureus* [12,13]. Their antibacterial activity can be attributed to inhibiting the bacterial ribosome, which has been supported by extensive biochemical and X-ray crystallographic studies [14].

Xenocoumacin, an antibiotic that is structurally-related to amicoumacin, has been identified in the Gram-negative bacterium *Xenorhabdus nematophila* [15]. *Xenorhabdus* species are mutualistic bacterial symbionts of insect-pathogenic (entomopathogenic) nematodes in the genus *Steinernema* [16,17]. The bacteria are carried in the nematode intestine during the infective juvenile developmental stage. Upon penetrating an insect host, the infective juvenile expels *Xenorhabdus* into the hemocoel of the host insect. The bacteria rapidly proliferate and secrete a variety of metabolites, immunomodulators, antibiotics, and cytotoxins to regulate interactions among its mutualist nematode host, its insect host prey, and its bacterial and fungal competitors in the decomposing insect carcass. The nematode reproduces inside the insect host, consuming the bacterial biomass, and new infective juveniles colonized by *Xenorhabdus* emerge from the insect cadaver and search for another meal [18]. During this process, xenocoumacin is thought to be the dominant antibiotic involved in sterilizing the insect environment for the specific *X. nematophila-Steinernema* pair [19]. In xenocoumacin biosynthesis, the biologically inactive prexenocoumacins containing an *N*-acyl-d-Asn side chain are first produced by the nonribosomal peptide synthetase-polyketide synthase hybrid pathway. The *N*-acyl-d-Asn is then cleaved by a dedicated peptidase in the pathway, leading to the primary active component xenocoumacin-1 [19]. A similar pro-drug activation mechanism has also been reported for the structurally-related amicoumacins in the marine *Bacillus subtilis* 1779 isolate [20]. However, the biological role of the *N*-acyl-d-Asn moieties (*N*-acylation) in the amicoumacins and xenocoumacins among other metabolites remains unclear at the mechanistic level.

Given the complexity of molecular interactions in the tripartite bacteria-nematode-insect system, the *Xenorhabdus* genus remains an attractive source for bioprospecting of specialized metabolites with pharmacalogical potential. Indeed, biologically active metabolites have been isolated from *Xenorhabdus* bacteria, including dithiolopyrrolone cytotoxins, indole antibacterial derivatives, glidobactin proteasome inhibitors, pristinamycin antibiotics, isocyanide invertebrate innate immunosuppressants, antifungals, and cyclic peptides among others [21,22,23,24]. Genomic analyses of individual *Xenorhabdus* species have revealed that the number of predicted biosynthetic gene clusters substantially exceeds the number of known metabolites isolated from this genus [25]. This disparity arises in part from the inability to activate many of the “orphan” biosynthetic gene clusters under standard laboratory culture protocols [26]. Hence, optimizing the bacterial culture conditions to mimic features of the insect environment is a useful strategy to enhance the production of novel bioactive metabolites [25,27].

Using a culture medium designed to mimic the amino acid content of waxmoth larval circulatory fluid (a *Galleria mellonella* “hemolymph mimetic medium”), we report the stimulation of the amicoumacin antibiotics in *X. bovienii*, a host not previously known to produce amicoumacins. This finding extends amicoumacin biosynthesis from known Gram-positive producers to a new Gram-negative producer. We further establish the gene cluster responsible for amicoumacin biosynthesis in *X. bovienii* through genome synteny and comparative gene cluster analysis with the amicoumacin pathway in *Bacillus subtilis* and the xenocoumacin pathway in *X. nematophila*. *X. bovienii* produces both amicoumacins and *N*-acetylated amicoumacins, the latter of which were not found in the corresponding sequenced *B. subtilis* isolate. This difference can be attributed to the presence of a non-syntenic predicted acetyltransferase found in the *X. bovienii* amicoumacin gene cluster. We confirm that this isolated acetyltransferase acetylates amicoumacin A in vitro to produce its corresponding *N*-acetyl-amicoumacin A, providing a biochemical rationale for the *N*-acetylation of the amicoumacin family. We further demonstrate that this biochemical reaction destroys antibacterial activity. Finally, through structural modeling studies, we explore the mechanistic impact of *N*-acylation on amicoumacin’s antibacterial mode of action.

## 2. Results and Discussion

### 2.1. Structural Identification of Amicoumacin Metabolites from Hemolymph-Mimetic Medium

Select secondary metabolic pathways in the *Photorhabdus* and *Xenorhabdus* genera are stimulated by molecules present in the insect hemolymph [25,27]. We made a “hemolymph-mimetic medium” (HMM) based on the concentrations of the 20 proteinogenic amino acids in the hemolymph of the larval insect host *Galleria mellonella* (35 g/L total amino acids, Table S1) [25]. Organic extractable metabolites from *X. bovienii* Moldova were compared from cultures grown in HMM, Lysogeny Broth (LB), and LB supplemented with high concentrations of l-proline (72.6 mM), a known free amino acid nutrient signal that enhances the production of some secondary metabolites in *Xenorhabdus* and *Photorhabdus* [25]. Five milliliters of each of these media were inoculated with a single colony of *X. bovienii* Moldova and grown under aerobic conditions (250 rpm) at 30 °C for two days. The clarified culture media were subsequently extracted with butanol, and the organic layers were collected and dried under reduced pressure. These crude extracts were analyzed by LC/ESI-MS. Analysis of the spectra revealed the presence of one distinct peak at *m*/*z* 449 [M + H]^+^ in HMM that was not observed in the LB culture medium. This peak was also observed in the crude extract of *X. bovienii* grown in LB supplemented with L-proline albeit at a lower level of production relative to HMM (Figure S1).

**Figure 1:**
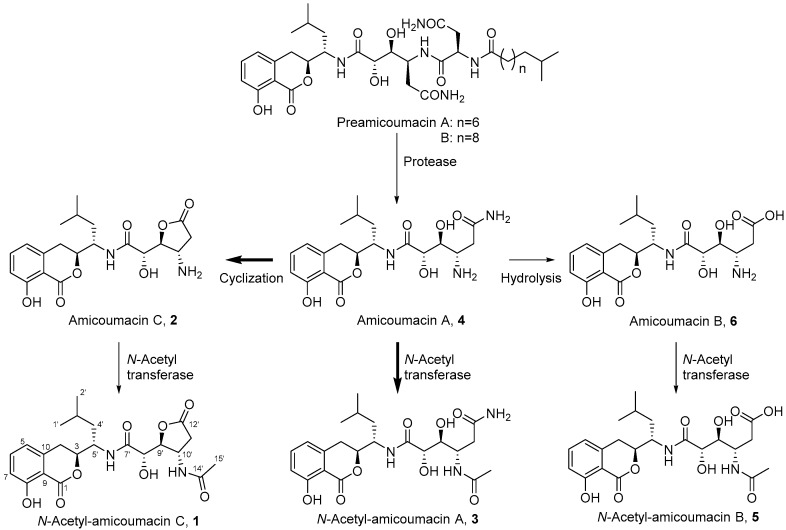
Amicoumacin structures **1**–**6** produced by *Xenorhabdus bovienii*. Preamicoumacins, which have been previously reported from *B. subtilis*, are the inactive pro-drug products that contain an *N*-acyl-d-Asn moiety. Our data suggest that amicoumacin A (**4**) is the dominant biosynthetic product in *X. bovienii* and the remaining metabolites are primarily derived from **4**. Bold arrows indicate transformations experimentally assessed in this study.

To structurally characterize the identified peak, a 6 L culture of *X. bovienii* Moldova was initiated in HMM. After 2 days of cultivation at 30 °C, the clarified medium was extracted with butanol and dried in vacuo to yield 1.2 g of crude material. The crude extract was subjected to multiple rounds of C_18_ high-performance liquid chromatography (HPLC), resulting in the purification of the target compound (0.7 mg). The pure compound was structurally characterized using 2D NMR (gCOSY, gHSQC, and gHMBC) and HR-ESI-QTOF-MS (Figures S2–S6). Interpretation of the spectral data revealed its identification as the previously reported bacterial metabolite *N*-acetyl-amicoumacin C (**1**) (Figure 1) [7]. The optical rotation value {${\left[\alpha \right]}_{D}^{25}$; −52.0 (*c* 0.1, CH_3_OH)} was consistent with that of reported data {${\left[\alpha \right]}_{D}^{23}$; −51.0 (*c* 0.4, CH_3_OH)}, supporting the same absolute configuration [7].

**Figure 2:**
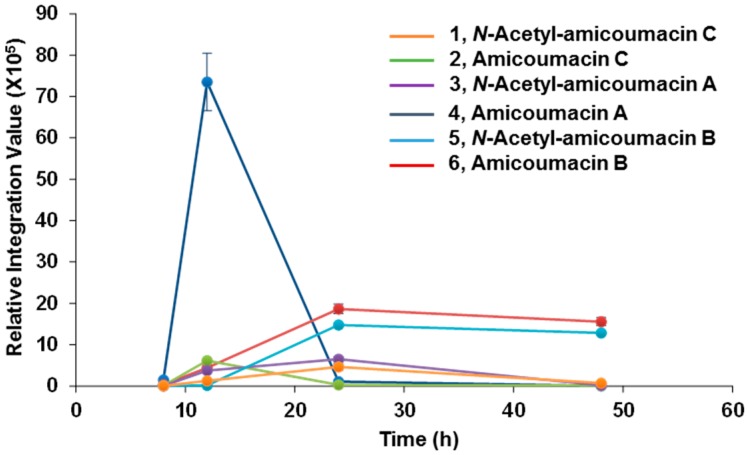
Time-course analysis of amicoumacin metabolites **1**–**6**.

With the identification of *N*-acetyl-amicoumacin C (**1**) in *X. bovienii*, we then explored the ability of *X. bovienii* to produce other members of the amicoumacin family in a time-dependent manner (Figure 2). A time-course analysis over 48 h in HMM revealed the production of five additional amicoumacin metabolites **2**–**6**, which were structurally determined by comparisons of the HR-ESI-QTOF-MS and MS/MS data to previous reports of these metabolites (Figures S7–S14). The structure of *N*-acetyl-amicoumacin A (**3**) purified from butanol extracts {${\left[\alpha \right]}_{D}^{25}$; −22.4 (*c* 0.05, CH_3_OH)} was confirmed by interpretation of ^1^H- and ^13^C-NMR spectra (Figures S15 and S16). Amicoumacin A (**4**), the major metabolite at earlier time points, was enriched by solid phase extraction (C_18_), purified via reversed-phase HPLC (C_18_), and structurally verified by 2D-NMR (Figures S17–S20). The optical rotation of amicoumacin A (**4**) {${\left[\alpha \right]}_{D}^{25}$; −69.0 (*c* 0.1, CH_3_OH)} was also consistent with that of literature value {${\left[\alpha \right]}_{D}^{25}$; −97.2 (*c* 1.0, CH_3_OH)} [3]. While the production of amicoumacin A (**4**) and amicoumacin C (**2**) were maximally observed at 12 h, they rapidly degrade within 24 h with a concomitant enhancement of the other amicoumacin derivatives **1**, **3**, **5** and **6**.

This inverse correlation in production is similar to that observed for the structurally-related xenocoumacins (Figure S21), which are dihydroisocoumarin antibiotic metabolites produced by *X. nematophila* [28]. This result suggests that amicoumacin A (**4**) is largely a precursor in the formation of or degradation to the other amicoumacin derivatives in *X. bovienii*. When we cultured *X. bovienii* in the direct presence of Amberlite XAD-7, which serves as a macroreticular resin to trap metabolites during cultivation, only amicoumacin A (**4**) was detected as the major metabolite over 48 h, further supporting its role as the central metabolite in *X. bovienii* amicoumacin biosynthesis (Figure S22). To better understand the structural relationships among the observed amicoumacin metabolites, we incubated pure amicoumacin A (**4**) in sterilized LB liquid medium and water in the absence of bacteria. Unexpectedly, formation of the intramolecular cyclization product amicoumacin C (**2**) was readily observed in the LB medium supplemented with amicoumacin A (**4**) (Figure S23). Cyclization was not observed in pure water under the conditions of our experiment, indicating that the medium components can catalyze degradative lactone formation.

### 2.2. Antibacterial Evaluation of Amicoumacins

Amicoumacins have been evaluated previously for antibacterial activities against a number of bacterial strains. It has been reported that amicoumacin A (**4**) exhibits significant antibacterial activities against Gram-positive bacteria, including *Bacillus subtilis* 1779 (MIC = 20.0 µg/mL), *Staphylococcus aureus* UST950701-005 (MIC = 5.0 µg/mL), and methicillin-resistant *Staphylococcus aureus* ATCC43300 (MIC = 4.0 µg/mL), in addition to a series of Gram-negative *Helicobacter pylori* strains (average of MIC values = 1.4 µg/mL) [8,13,20,29]. In contrast, structurally related amicoumacins B (**6**), C (**2**), and *N*-acetylamicoumacin C (**1**) were inactive at the concentration of 100 µg/mL or 1 mg/mL [20,29]. Preamicoumacins (Figure 1), the pro-drug forms, are similarly inactive against *B. subtilis* 1779 (MIC ≥ 100 µg/mL) and *S. aureus* UST950701-005 (MIC ≥ 100 µg/mL) [20]. To determine if *N*-acetylation directly attenuates the antibacterial activity of amicoumacin A, we examined amicoumacin A (**4**) and *N*-acetylamicoumacin A (**3**) activities against the model Gram-positive strain, *Bacillus subtilis* BR151. Our results showed that amicoumacin A (**4**) possesses inhibitory activity against *B. subtilis* BR151 at 100 µg/mL as expected, while *N*-acetyl amicoumacin A (**3**) was inactive (MIC ≥ 200 µg/mL) (Figure S24), indicating that *N*-acetylation serves as an amicoumacin resistance mechanism.

### 2.3. Amicoumacin Biosynthetic Gene Cluster Analysis

**Figure 3:**
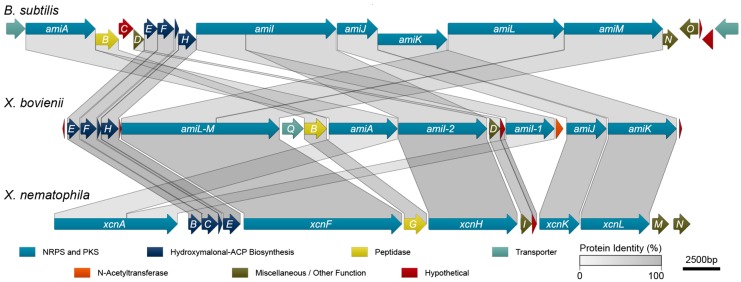
Synteny of biosynthetic gene clusters for amicoumacins in *X. bovienii* Moldova, amicoumacins in *B. subtilis* subsp. inaquosorum KCTC 13429, and xenocoumacins in *X. nematophila* ATCC 19064.

To identify the genetic determinants of amicoumacin biosynthesis in *X. bovienii*, we performed an antiSMASH analysis [30] on the genome of *X. bovienii* Moldova. This analysis revealed a putative biosynthetic pathway with high homology to the xenocoumacin biosynthetic pathway from *X. nematophilia.* Subsequent protein sequence homology analysis utilizing conserved domain database comparisons, BLAST, and pairwise sequence alignments among the sequenced amicoumacin/xenocoumacin producers, *X. bovienii*, *B. subtilis*, and *X. nematophila,* revealed that *X. bovienii* possesses homologs of *amiA*, *amiB*, and *amiD* through *amiM* from *B. subtilis* subsp. inaquosorum KCTC 13429 (Figure 3, Table 1). However, several of the genes are out of biosynthetic “order” compared to the collinear arrangement in *B. subtilis* subsp. inaquosorum KCTC 13429. In *X. bovienii*, the *amiI* gene is split across two genes (annotated here as *amiI-1* and *amiI-2* based on functional domain homology), while *amiL* and *amiM* appear to have fused into one gene (denoted as single gene *amiL-M* in *X. bovienii*, Figure 3).

The *X. bovienii* amicoumacin gene cluster also lacks *amiC* (a hypothetical protein), *amiN* (a putative kinase), and *amiO* (an alkaline phosphatase). The *X. bovienii* amicoumacin gene cluster shares homology to 11 of the xenocoumacin biosynthesis genes, *xcnB* through *xcnL*, from *X. nematophila* ATCC 19061 (Figure 3). The genes are syntenic across both *Xenorhabdus* strains, and while *X. bovienii* possesses a homolog of *xcnA*, it is specifically split across two genes including *amiA* and *amiI-1* (Figure 3). Other key differences compared to the xenocoumacin gene cluster include the deletion of *xcnM*, a saccharopine dehydrogenase, and *xcnN*, a fatty acid desaturase. Lastly, the amicoumacin biosynthetic pathway in *X. bovienii* Moldova harbors additional genes, such as *amiP*, *amiQ*, and *amiS* relative to the *B. subtilis* and *X. nematophila* pathways. Bioinformatic analysis revealed that *amiP* is a hypothetical protein, *amiQ* is homologous to multidrug membrane transporters, and *amiS* shares homolgy to *N*-acetyltransferases (Table 1). BLASTP analysis of AmiS demonstrated the existence of numerous closely related homologs in important bacteria, such as *Vibrio cholerae*, *Vibrio parahaemolyticus*, and *Vibrio vulnificus* (Table S2). Presence of the *N*-acetyltransferase AmiS suggested that it could account for amicoumacin A resistance through the formation of the inactive *N*-acetyl amicoumacin derivatives detected in HMM.

The amicoumacins and xenocoumacins, with their considerable similarities in structures and biosynthetic pathways, can be considered as examples along the spectrum of a single class of dihydroisocoumarin metabolites. *X. nematophila* is thought to have acquired many of the genes for xenocoumacin biosynthesis via horizontal gene transfer from *Bacillus* spp [31]. Here, we hypothesize that *X. bovienii* Moldova likewise acquired the amicoumacin biosynthetic gene cluster via horizontal gene transfer, albeit from an unknown source. Acquisition via horizontal gene transfer is supported by the fact that the gene cluster in *X. bovienii* Moldova is flanked by transposases. Interestingly, the biosynthetic gene clusters in both *B. subtilis* and *X. nematophila* display a higher degree of collinearity than does the amicoumacin biosynthetic gene cluster in *X. bovienii* Moldova. This could reflect a more recent acquisition of the gene cluster in the latter, as Callahan et al. have suggested that collinearity tends to arise spontaneously over time. Lastly, natural *N*-acetyl-amicoumacins have been identified from other marine-derived *Bacillus* sources [32], and these strains likely also contain a dedicated *N*-acetyltransferase.

### 2.4. In Vitro N-Acetylation of Amicoumacin A

*N*-acylation plays a critical role in controlling the biological activity of the amicoumacin family. Biologically inactive preamicoumacins undergo a pro-drug activation mechanism whereby the *N*-acyl-d-Asn is cleaved off by the peptidase AmiB to generate the biologically active form [20]. In contrast, we show that *N*-acetylation at the same site in amicoumacin A serves to attenuate its function. In *X. nematophila*, the xenocoumacins are alternatively detoxified by the producing host through an XcnMN-catalyzed oxidative cleavage of its guanidino side chain [28,33]. Consistent with differing detoxification mechanisms across the two *Xenorhabdus* strains, amicoumacin lacks the guanidino functional group, and the *X. bovienii* amicoumacin pathway correspondingly lacks the XcnMN homologs as expected. To confirm the predicted function of *X. bovienii* AmiS in amicoumacin biosynthesis, we examined the ability of AmiS to catalyze the conversion of amicoumacin A (**4**) into *N*-acetyl-amicoumacin A (**3**).

**Figure 4:**
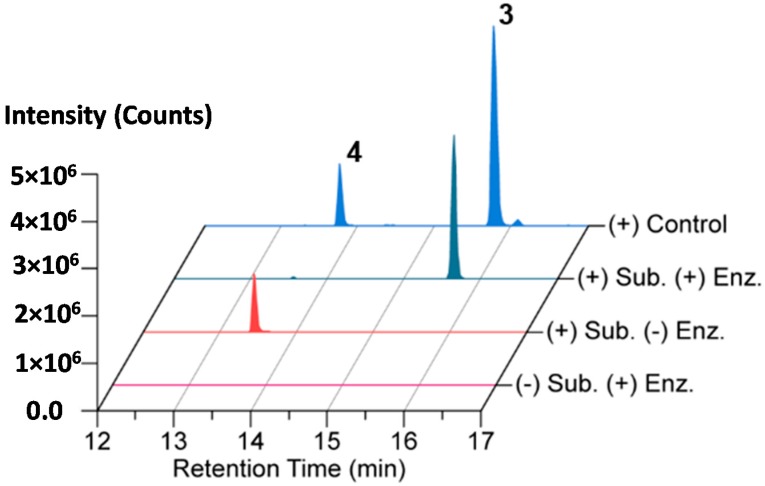
Conversion of amicoumacin A (**4**) into *N*-acetyl-amicoumacin A (**3**) by in vitro *N*-acetylation catalyzed by AmiS.

AmiS from *X. bovienii* str. feltiae Moldova was cloned, overexpressed, and purified as an *N*-terminal hexa-histidine-tagged protein for biochemical analysis (Figure S25). An in vitro biochemical reaction mixture containing isolated amicoumacin A (**4**) as the substrate and acetyl-CoA as the acyl donor was assembled, and the reaction was initiated with AmiS. The reaction proceeded at 25 °C for 1 h followed by an incubation at 12 °C for 5 h before being quenched via extraction with an equal volume of ethyl acetate. The organic fraction was dried and the material was analyzed by LC-HR-ESI-QTOF-MS and MS/MS. The LC/MS and tandem MS chromatograms support the formation of *N*-acetyl-amicoumacin A (**3**) in the presence of AmiS with near quantitative conversion under the reaction conditions (Figure 4 and Figure S26).

### 2.5. Ribosomal Structural Modeling of N-Acetyl-Amicoumacin A

**Figure 5:**
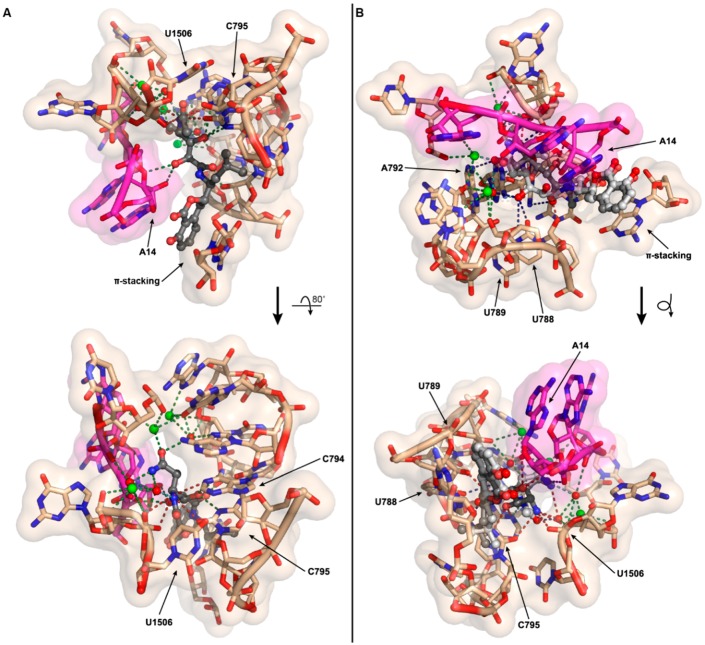
Computational models of *N*-acetyl amicoumacin A ribosomal interactions. (**A**) The *N*-acetyl group (fixed amicoumacin A backbone) was energy minimized in the presence of the ribosomal binding pocket. The minimal energy conformation demonstrates steric clashes between the *N*-acetyl group and the ribosome at U1506 and C795, as well as a net loss of three polar interactions relative to amicoumacin A; (**B**) The complete *N*-acetyl amicoumacin A molecule (dark grey) was energy minimized in the presence of the ribosomal binding pocket. The molecule adopts an altered binding conformation relative to amicoumacin A (light grey) resulting in a reorganization of polar interactions. For both (**A**,**B**), predicted polar contacts are indicated by dotted lines: red, interaction is lost; green, interaction is maintained; light blue, interaction is maintained but spatially altered; and dark blue, interaction is formed.

As the antibacterial mechanism of action for amicoumacin A has already been established structurally and biochemically by the Sergiev, Steitz, and Mankin labs [14], we explored how *N*-acylation of amicoumacin could disrupt its native function. Amicoumacin A functions as an antibacterial through its ability to bind to the ribosome and disrupt protein synthesis. Unlike many antibiotics, which target the peptidyl transferase center or the exit tunnel, amicoumacin A binds in the E-site stabilizing the E-tRNA–mRNA interactions, inhibiting ribosomal translocation. Utilizing the 2.5 Å crystal structure of amicoumacin A in complex with the *T. thermophilus* ribosome [14], we appended on the *N*-acetyl moiety in an energy minimized conformation and subsequently conducted further energy minimization of *N*-acetyl amicoumacin A in the presence of the ribosome to assess potential alternative binding conformations (Figure 5A). When the amicoumacin backbone is fixed and the acetyl group alone is energy minimized in the presence of a fixed ribosomal binding pocket, steric clashes with the surrounding U1506 and C795 residues of the 16S rRNA are observed, and all predicted polar interactions occurring between the primary amine of amicoumacin A and the ribosome are eliminated, except for the interaction of the nitrogen and a water molecule participating in a larger bonding network (Figure 5A). In this conformation, the carbonyl of the acetyl group additionally forms a contact with the water molecule. When *N*-acetyl amicoumacin A is minimized as a flexible molecule in a ridged ribosomal binding pocket, the ring system undergoes minimal alteration, but the linear portion of the molecule undergoes larger conformational shifts ranging from approximately 1 to 3 Å (Figure 5B). This conformational change corresponds to a broad reorganization of the polar contact network. In all of the predicted conformations, the loss of polar contacts with the ribosome likely serves to destabilize *N*-acetyl amicoumacin A binding. Docking of the larger preamicoumacin with its *N*-acyl-d-Asn moiety was not attempted due to the clear steric clashes that would occur with the ribosomal structure, supporting a steric ribosomal exclusion mechanism for the preamicoumacins. These models taken in concert with current pro-drug activation phenotypes and minimal inhibitory concentration data suggest that that the free- versus acylated-4-amino group plays a critical structural role in modulating the functionality and potency of the amicoumacin family.

## 3. Materials and Methods

### 3.1. General Procedures

Optical rotations were measured on a P2010 polarimeter (JASCO Inc, Easton, MD, USA). Low-resolution electrospray ionization (ESI) mass spectra were measured on a 6120 Quadrupole HPLC/MS system (Agilent, Santa Clara, CA, USA) equipped with a Kinetex C_18_ (2) 5 μm column (4.6 mm × 250 mm, Phenomenex, Torrance, CA, USA). High-resolution ESI-MS data were obtained using an Agilent iFunnel 6550 Q-TOF (quadrupole time-of-flight) MS instrument. NMR spectra including 2D-NMR (gCOSY, gHSQC, and gHMBC) were recorded on an Agilent 600 MHz NMR spectrometer equipped with a cold probe. Chemical shifts were referenced to residual solvents. A Sep-Pak^®^ Vac 35cc (10 g) C_18_ cartridge (Waters, Milford, MA, USA) was used for flash column chromatography. The separation of crude extracts was performed on an Agilent Prepstar HPLC system using an Agilent Polaris C_18_-A 5 μm (21.2 mm × 250 mm) column and a Phenomenex Luna C_18_ or C_8_ (2) 10 μm column (10.0 mm × 250 mm).

### 3.2. Bacterial Strain, Growth Condition and Analytical-Scale Cultivation

*X. bovienii* str. feltiae Moldova strain was generously provided by Dr. Heidi Goodrich-Blair (University of Wisconsin-Madison, Madison, WI, USA). *X. bovienii* Moldova was routinely grown at 30 °C in lysogeny broth (LB; 1% (*w*/*v*) tryptone, 0.5% (*w*/*v*) yeast extract, 1% (*w*/*v*) NaCl) or on LB agar plates (LB with 1.5% (*w*/*v*) agar). Hemolymph-mimetic medium (HMM) was designed based on the concentrations of the 20 proteinogenic amino acids in the hemolymph of *Galleria mellonella* larvae (35 g/L total amino acids, 5 g/L Yeast extract) [25]. For metabolite profiling, a single colony of *X. bovienii* Moldova grown on a LB agar plate was inoculated into 5 mL each of three media: LB, LB supplemented with l-proline (72 mM), and HMM; and then grown at 30 °C and 250 rpm for 48 h. The cultures were centrifuged (2000× *g,* 15 min, 4 °C) and the supernatants were extracted with *n*-butanol (1 × 5 mL). The organic layer was dried under reduced pressure on a HT-4X system (Genevac Inc, Gardiner, NY, USA) for 2 h. The crude extract was then resuspended in 100 μL of methanol and analyzed via single quadrupole LC/MS (Injection Volume: 10 μL; Column: Phenomenex Kinetex C_18_ (100 Å) 5 μm (250 mm × 4.6 mm) column; Flow Rate: 0.7 mL/min; Mobile phase composition: 10 to 100% aqueous acetonitrile containing 0.1% formic acid over 30 min).

### 3.3. Time-Course Analysis of Amicoumacin Production

A single colony of *X. bovienii* Moldova was inoculated into 5 mL of HMM and cultured overnight at 30 °C and 250 rpm. The following day, four, 5 mL aliquots of HMM were inoculated at 1:1000. These cultures were incubated at 30 °C and 250 rpm; and subsequently extracted with butanol at four different time points: 8, 12, 24, or 48 h after inoculation. The metabolite profiles were analyzed in the same manner as described in Section 3.2. The time-course experiment was performed in triplicate.

### 3.4. Larger-Scale Cultivation and Extraction

To extract *N*-acetylamicoumacin C (**1**) and *N*-acetylamicoumacin A (**3**), *X. bovienii* Moldova was grown overnight in LB (30 °C, 250 rpm). 25 μL of culture was then used to inoculate six, 5 mL LB cultures for overnight growth at 30 °C and 250 rpm. Each of the 5 mL cultures was transferred to one of six, 4 L Erlenmeyer flasks containing 1 L of HMM and cultivated at 30 °C and 250 rpm. After 2 days, the 6 L culture was pooled and centrifuged at 14,000× *g* for 20 min, and the clarified supernatant was extracted twice with an equal volume of butanol (12 L total) followed by concentration under reduced pressure to yield 1.2 g of crude material. For the extraction of amicoumacin A (**4**), the above process was repeated; however, sterilized XAD-7 resin (20 g/L) was added to the 6 × 1 L HMM growths. After 2 days of cultivation, the XAD-7 resin was collected via filtration using cheese cloth. The resin was extracted with methanol and acetone, and the organic fraction was dried under reduced pressure, yielding 1.0 g of crude material.

### 3.5. Isolation of Amicoumacin Metabolites ***1***, ***3*** and ***4***

The crude extract containing *N*-acetylamicoumacin C (**1**) was fractionated by reversed-phase C_18_ flash column chromatography, eluting with a step gradient from 20% methanol in water to 100% methanol to give five fractions (20%, 40%, 60%, 80%, and 100% methanol). Single quadrupole LC/MS analysis of the fractions (following the method detailed in Section 3.2. Bacterial Strain, Growth Condition and Analytical-Scale Cultivation) revealed that **1** was in the 80% methanol fraction. The 80% methanol fraction was dried and subjected to reversed-phase HPLC separation on an Agilent Prepstar HPLC system (Agilent Polaris C_18_-A 5 μm (21.2 mm × 250 mm) column) with a linear gradient elution (10% aqueous acetonitrile–100% aqueous acetonitrile in 0.01% trifluoroacetic acid (TFA) over 60 min; Flow Rate: 8 mL/min; 1 min fraction collection interval). **1** eluted in fraction 27. Additional reversed-phase HPLC separation of fraction 27 (Phenomenex Luna C_18_ column, 5 μm, 250 mm × 10.0 mm column) under isocratic conditions (40% aqueous acetonitrile containing 0.01% TFA over 15 min; Flow Rate: 4 mL/min) led to the purification of **1** (0.7 mg) at 9.4 min. Compound **3** present in the 60% methanol fraction from the Waters Sep-Pak^®^ Vac 35cc (10 g) C_18_ cartridge was isolated using an Agilent Prepstar HPLC system (Agilent Polaris C_18_-A 5 μm (21.2 mm × 250 mm) column) with a gradient elution (10% aqueous acetonitrile–70% aqueous acetonitrile for 60 min; Flow Rate: 10 mL/min; 1 min fraction window). **3** was then purified from fraction 30 by reversed-phase HPLC (Phenomenex Luna C_18_ (2) (100 Å) 10 μm (10.0 × 250 mm) column, Flow rate; 4 mL/min) using a gradient elution (10%–100% aqueous acetonitrile in 0.01% TFA for 60 min) leading to 0.5 mg of product with a retention time of 25.8 min.

The crude material from the XAD-7 extract containing amicoumacin A (**4**) was fractionated by a Waters Sep-Pak^®^ Vac 35cc (10 g) C_18_ cartridge with a step gradient from 40%–100% methanol in water (40%, 60%, 80% and 100% methanol). The 60% methanol fraction contained **4** and was subjected to reversed-phase HPLC separation on an Agilent Prepstar HPLC system (Agilent Polaris C_18_-A 5 μm (21.2 mm × 250 mm) column) with a linear gradient elution (10%–100% aqueous methanol over 60 min; Flow Rate: 8 mL/min; 1 min fraction collection interval). **4** was isolated from a combined fraction (24 + 25) by reversed-phase HPLC (Phenomenex Luna C_18_ (2) (100 Å) 10 μm (10.0 mm × 250 mm) column, Flow rate; 4 mL/min) with a gradient elution (10%–100% aqueous acetonitrile for 60 min). **4** (*t*_R_ = 15.3 min, 1.2 mg) was then purified by reversed-phase HPLC (Phenomenex Luna C_8_ (2) (100 Å) 10 μm (10.0 × 250 mm) column; Flow Rate: 4 mL/min) with a gradient elution (10% to 100% aqueous acetonitrile containing 0.01% TFA).

*N-Acetylamicoumacin C* (**1**): colorless oil; ${\left[\alpha \right]}_{D}^{25}$; −52.0 (*c* 0.10, CH_3_OH); ^1^H-NMR (methanol-*d*_4_) δ 7.45 (1H, t, *J* = 8.0 Hz, H-6), 6.83 (1H, d, *J* = 8.0 Hz, H-7), 6.79 (1H, d, *J* = 8.0 Hz, H-5), 4.76 (1H, t, *J* = 2.4 Hz, H-9′), 4.67 (1H, dt, *J* = 11.3, 3.8 Hz, H-3), 4.49 (1H, dt, *J* = 8.9, 2.5 Hz, H-10′), 4.41 (1H, d, *J* = 2.4 Hz, H-8′), 4.30 (1H, dt, *J* = 10.9, 3.9 Hz, H-5′), 3.03 (1H, dd, *J* = 18.3, 8.9 Hz, H-11′), 2.95 (2H, m, H-4), 2.35 (1H, dd, *J* = 18.3, 2.7 Hz, H-11′), 1.85 (3H, s, H-15′), 1.82 (1H, m, H-4′), 1.62 (1H, m, H-3′), 1.41 (1H, ddd, *J* = 13.9, 9.8, 4.2 Hz, H-4′), 0.95 (3H, d, *J* = 6.7 Hz, H-1′), 0.88 (3H, d, *J* = 6.5 Hz, H-2′); ^13^C-NMR (methanol-*d*_4_) δ 176.4 (C-12′), 172.4 (C-14′), 171.4 (C-7′), 169.5 (C-1), 161.7 (C-8), 139.8 (C-10), 136.0 (C-6), 118.0 (C-5), 115.2 (C-7), 107.9 (C-9), 85.8 (C-9′), 81.1 (C-3), 71.8 (C-8′), 49.0 (C-5′), 46.6 (C-10′), 39.0 (C-4′), 35.9 (C-11′), 29.4 (C-4), 24.2 (C-3′), 22.3 (C-1′), 20.8 (C-15′), 20.3 (C-2′); HR-ESI-QTOF-MS [M + H]^+^* m*/*z* 449.1919 (calcd. for C_22_H_29_N_2_O_8_, 449.1924).

*N-Acetylamicoumacin A* (**3**): colorless oil; ${\left[\alpha \right]}_{D}^{25}$; −22.4 (*c* 0.05, CH_3_OH); ^1^H-NMR (methanol-*d*_4_) δ 7.43 (1H, t, *J* = 8.0 Hz, H-6), 6.82 (1H, d, *J* = 8.0 Hz, H-7), 6.78 (1H, d, *J* = 8.0 Hz, H-5), 4.66 (1H, dt, *J* = 12.7, 2.9 Hz, H-3), 4.47 (1H, dt, *J* = 9.0, 4.6 Hz, H-10′), 4.33 (1H, dt, *J* = 10.6, 3.7 Hz, H-5′), 4.01 (1H, d, *J* = 6.4 Hz, H-8′), 3.79 (1H, m, H-9′), 3.13 (1H, dd, *J* = 16.4, 12.7 Hz, H-4), 2.89 (1H, dd, *J* = 16.5, 2.9 Hz, H-4), 2.63 (1H, dd, *J* = 15.1, 4.3 Hz, H-11′), 2.48 (1H, dd, *J* = 15.1, 8.8 Hz, H-11′), 1.93 (3H, s, H-15′), 1.81 (1H, ddd, *J* = 13.9, 10.8, 4.8 Hz, H-4′), 1.73 (1H, m, H-3′), 1.46 (1H, ddd, *J* = 13.6, 9.3, 4.2 Hz, H-4′), 0.97 (3H, d, *J* = 6.7 Hz, H-1′), 0.94 (3H, d, *J* = 6.5 Hz, H-2′); ^13^C-NMR (methanol-*d*_4_) *δ* 175.2 (C-12′), 173.6 (C-7′), 171.7 (C-14′), 169.7 (C-1), 161.7 (C-8), 140.3 (C-10), 136.0 (C-6), 118.1 (C-5), 115.2 (C-7), 108.0 (C-9), 81.4 (C-3), 74.1 (C-9′), 72.1 (C-8′), 50.0 (C-5′), 48.6 (C-10′), 39.4 (C-4′), 34.7 (C-11′), 29.4 (C-4), 24.3 (C-3′), 22.3 (C-1′), 21.3 (C-15′), 20.6 (C-2′); HR-ESI-QTOF-MS [M + H]^+^ *m*/*z* 466.2185 (calcd. for C_22_H_32_N_3_O_8_, 466.2189).

*Amicoumacin A* (**4**): colorless oil; ${\left[\alpha \right]}_{D}^{25}$; −69.0 (*c* 0.10, CH_3_OH); ^1^H-NMR (DMSO-*d*_6_) *δ* 10.80 (1H, s, 8-OH), 7.78 (1H, m, 6′-NH), 7.78 (2H, m, 10′-NH_2_), 7.61 (1H, s, 12′-NH_2_), 7.47 (1H, t, *J* = 8.0 Hz, H-6), 7.19 (1H, s, 12′-NH_2_), 6.84 (1H, d, *J* = 8.0 Hz, H-7), 6.80 (1H, d, *J* = 8.0 Hz, H-5), 5.94 (1H, brs, 8′-OH), 5.54 (1H, d, *J* = 5.0 Hz, 9′-OH), 4.69 (1H, m, H-3), 4.18 (1H, m, H-5′), 3.97 (1H, d, *J* = 6.5 Hz, H-8′), 3.86 (1H, d, *J* = 2.3 Hz, H-9′), 3.49 (1H, m, H-10′), 3.00 (1H, dd, *J* = 16.4, 12.6 Hz, H-4), 2.85 (1H, dd, *J* = 16.6, 2.9 Hz, H-4), 2.65 (1H, dd, *J* = 17.1, 2.9 Hz, H-11′), 2.36 (1H, dd, *J* = 17.0, 10.0 Hz, H-11′), 1.67 (1H, ddd, *J* = 13.4, 11.0, 4.4 Hz, H-4′), 1.61 (1H, m, H-3′), 1.31 (1H, ddd, *J* = 13.3, 9.5, 3.9 Hz, H-4′), 0.88 (3H, d, *J* = 6.6 Hz, H-1′), 0.83 (3H, d, *J* = 6.5 Hz, H-2′); ^13^C-NMR (DMSO-*d*_6_) *δ* 172.6 (C-12′), 172.5 (C-7′), 169.5 (C-1), 161.2 (C-8), 140.9 (C-10), 136.7 (C-6), 118.9 (C-5), 115.7 (C-7), 108.7 (C-9), 81.4 (C-3), 72.2 (C-8′), 71.2 (C-9′), 49.9 (C-10′), 48.6 (C-5′), 39.4 (C-3′), 32.1 (C-11′), 29.4 (C-4), 24.3 (C-4′), 23.7 (C-1′), 21.9 (C-2′); HR-ESI-QTOF-MS [M + H]^+^ *m*/*z* 424.2083 (calcd. for C_20_H_30_N_3_O_7_, 424.2084).

### 3.6. Minimum Inhibitory Concentration Determination of Amicoumacin A and N-Acetylamicoumacins

Amicoumacin A (**4**), *N*-acetylamicoumacin A (**3**), and *N*-acetylamicoumacin C (**1**) were evaluated for antibacterial properties against the Gram-positive bacterium *Bacillus subtilis* BR151. *B. subtilis* BR151 was grown on an LB agar plate at 30 °C overnight. A single, well-defined colony of the bacterium was inoculated into 5 mL of LB and grown overnight in a shaking incubator (30 °C, 250 rpm). The overnight outgrowth was then subcultured 1:1000 into 5 mL of fresh LB and allowed to grow until OD_600_ = 0.1. This culture was then diluted 1:1000 into LB. Compounds **1**, **3**, and **4** were prepared in DMSO to a concentration of 10 µg/µL. Ampicillin was also prepared in this manner as a positive control. 50 µL of LB was dispensed into each well of a 96-well plate less the first column to which 96 µL of LB was added. 4 µL of the desired compound was added to the first well to give a final concentration of 400 µg/mL. A serial dilution was initiated from the first well. 50 µL of the diluted bacterial culture was then added to each well resulting in a maximum concentration of 200 µg/mL (100 µL final volume). DMSO was used as a vehicle control. All experimental samples were tested in triplicate, less the DMSO vehicle control, which was tested in duplicate. The plates were sealed and incubated at 30 °C overnight. Measurement of bacterial growth inhibition was assessed by OD_600_ values. The MIC was determined as the compound concentration that inhibited cell growth during the incubation period.

### 3.7. Identification of the Amicoumacin Biosynthetic Pathway in X. bovienii Moldova

The *Xenorhabdus bovienii* Moldova genome was analyzed using antiSMASH [30]. A putative biosynthetic gene cluster was identified based on annotated high homology to the xenocoumacin biosynthetic pathway from *X. nematophilia*. The functional domains of each protein encoded by the biosynthetic gene cluster were predicted using the NCBI Conserved Domain Database (CDD) server in conjunction with BLASTp searches. For nonribosomal peptide synthetases (NRPSs), the specificities of the adenylation domains were predicted using the online program NRPSpredictor2 [34].The functional domains of each of the proteins were then compared qualitatively with those of the proteins AmiA-M from *Bacillus subtilis* subsp. inaquosorum KCTC 13429, which have been reported to possess high similarity (>97% Identity, >98% similarity) to the known producer *Bacillus subtilis* 1779 whose sequence is presently unavailable on GenBank. The amino acid sequences were also compared using the EMBL-EBI Pairwise Sequence Alignment Tool, EMBOSS Water [35]. The same comparisons were made with the xenocoumacin-synthesizing proteins XcnA-L from *X. nematophila* ATCC 19064. The genes in the *X. bovienii* Moldova cluster were renamed here according to their homology with the amiA-M genes in *B. subtilis* 1779, with additional genes being named in alphabetical order from upstream to downstream.

### 3.8. Construction of 6×His-Tagged AmiS

Using the primers NTHisNdeI and EndNTXhoI, *amiS* was amplified via PCR. Reactions (50 μL) containing 5% (*v*/*v*) dimethyl sulfoxide (DMSO) and 1 μL of confluent *X. bovienii* Moldova as template were run using Phusion^®^ High-Fidelity DNA Polymerase (NEB, Ipswich, MA, USA) according to the manufactuer’s protocol. A C1000 Touch™ thermal cycler equipped with a Dual 48/48 Fast Reaction Module (Bio-Rad, Hercules, CA, USA) was used to thermal cycle as follows: 98 °C, 8 min; 98 °C, 10 s, 72–55 °C gradient, 15 s, 72 °C, 15 s × 34 cycles; 72 °C, 5 min; 12 °C, ∞. Reactions were assessed on a 0.75% agarose gel in 1 × TAE (40 mM tris pH 7.6, 20 mM acetic acid, and 1 mM EDTA) stained with GelGreen™ (Biotium, Hayward, CA, USA). Amplicons were purified using the QIAquick PCR Purification Kit (Qiagen, Hilden, Germany) according to the manufacturer’s protocol, and the products were pooled. pET28a(+) (EMDMillipore-Novagen, Darmstadt, Germany) was prepared via the QIAprep Spin Miniprep Kit (Qiagen) according to the manufacturer’s protocol. pET28a(+) was chosen as the cloning vector as it afforded facile incorporation of a N-terminal 6×His tag and thrombin cleavage site. Restriction digestion was carried out using NdeI and XhoI (NEB) on both the purified PCR product as well as the pET28a(+) plasmid. Reactions were purified using the QIAquick PCR Purification Kit according to the manufacturer’s protocol. Ligation was performed at a molar ratio of 1:3 vector:insert using T4 DNA ligase (NEB). Reactions were allowed to proceed for 15 min at room temperature. 4 μL of the ligation reaction was then directly transformed into 50 µL of MAX Efficiency^®^ DH5α™ chemically competent cells (Invitrogen, Carlsbad, CA, USA) following the manufacturer’s protocol with recovery occurring in 200 µL super optimal broth with catabolite repression (SOC, 2% (*w*/*v*) tryptone, 0.5% (*w*/*v*) yeast extract, 10 mM NaCl, 2.5 mM KCl, 10 mM MgCl_2_, 10 mM MgSO_4_, and 20 mM glucose). 150 µL of the transformation outgrowth was then plated onto LB agar plates supplemented with 25 µg/mL kanamycin (American Bioanalytical, Natick, MA, USA) and grown overnight at 37 °C to select for successful transformants. Colony PCR (cPCR) was then used to screen selected, single, well-defined colonies for successful gene insertion. cPCR reactions were carried out using the standard T7 promoter and T7 terminator primers along with Phusion^®^ High-Fidelity DNA Polymerase (NEB) according to the manufactuer’s protocol with the inclusion of 5% (*v*/*v*) DMSO. Thermal cycling was performed as follows: 98 °C, 8 min; 98 °C, 10 s, 55 °C, 15 s, 72 °C, 15 s × 34 cycles; 72 °C, 5 min; 12 °C, ∞. Amplicons were assessed on a 0.75% agarose gel in 1×TAE stained with GelGreen™ (Biotium, Hayward, CA, USA). Colonies bearing the gene of interest were then grown overnight at 37 °C and 250 rpm as suspension cultures in 5 mL LB supplemented with 25 µg/mL kanamycin. Plasmids were purified using the QIAprep Spin Miniprep Kit (Qiagen) according to the manufacturer’s protocol. The inserted gene along with proximal transcriptional and translational control elements was fully sequence validated (Genewiz, South Plainfield, NJ, USA). A single validated plasmid was chosen and termed pEAmiS.

### 3.9. Preparation of pEAmiS Expression Strains

Chemically competent *Escherichia coli* BAP1 (50 μL) was prepared according to standard methods and subsequently transformed by heat shock (42 °C, 45 s; 0 °C, 2 min) with 1 μL of pEAmiS. Cells were recovered for 1 h in 200 μL SOC at 37 °C and 250 rpm. Positive transformants were selected via overnight growth at 37 °C on LB agar plates supplemented with 25 μg/mL kanamycin. A single, well-defined colony was then chosen for overnight suspension culture in 5 mL of LB supplemented with 25 μg/mL kanamycin. This growth was used to inoculate a larger scale growth for protein purification (see Section 3.10. Overexpression, Isolation, and Purification of 6 × His-AmiS). Glycerol stocks were also prepared for long-term storage.

### 3.10. Overexpression, Isolation, and Purification of 6 × His-AmiS

1 L of Terrific Broth (TB; 1.2% (*w*/*v*) tryptone, 2.4% (*w*/*v*) yeast extract; 0.4% (*v*/*v*) glycerol) was inoculated at 1:1000 from a confluent culture of *E. coli* BAP1 harboring pEAmiS. The culture was grown at 37 °C and 250 rpm until the optical density (OD_600_) reached 0.5–0.6. The culture was then cooled on ice to slow growth and induced with isopropyl-l-thio-β-d-galactopyranoside (IPTG, American Bioanalytical) to a final concentration of 0.1 mM. Protein expression proceeded at 16 °C and 250 rpm for 20 h. The cell mass was collected via centrifugation at 12,000× *g* for 30 min at 4 °C in pre-massed centrifuge containers. Cells were massed, snap frozen in liquid nitrogen, and subsequently thawed on ice aided by the addition of chilled (4 °C) native lysis buffer (NLB; 1 mL/g wet cell mass; 50 mM NaH_2_PO_4_ pH 8, 300 mM NaCl, 2.5 mM imidazole) containing protease inhibitors (cOmplete™, EDTA-free Protease Inhibitor Cocktail, Roche, Basel, Switzerland). The cell pellet was resuspended to homogeneity, and lysozyme (1 mg/mL; American Bioanalytical) and DNase (5 μg/mL; Roche) were added. The mixture was incubated at room temperature and 80 rpm for 20 min. Sonication was then carried out on ice for 2 min with 10 s bursts follows by 30 s of recovery at 50% power using a FB-120 Sonic Dismembrator (ThermoFisher, Waltham, MA). Insoluble material was then removed by centrifugation at 30,000× *g* for 30 min at 4 °C. The supernatant was immediately mixed with 0.3 mL (CV) of NiNTA agarose resin (Qiagen) pre-equilibrated with native lysis buffer and allowed to batch bind on a rotisserie rotator at 4°C for 1 h. This mixture was then applied to a gravity column, and the clarified lysate was allowed to drain. The resin was then thoroughly washed twice with 15 mL wash buffer 20 (WB20, 50 mM NaH_2_PO_4_ pH 8, 300 mM NaCl, 20 mM imidazole) followed with two washes of 1.5 mL wash buffer 50 (WB50, 50 mM NaH_2_PO_4_ pH 8, 300 mM NaCl, 50 mM imidazole). The protein was eluted off of the resin in three stages. Two 1.5 mL volumes of elution buffer 100 (EB100; 50 mM NaH_2_PO_4_ pH 8, 300 mM NaCl, 100 mM imidazole) comprised the first step. This was followed by a 1.5 mL elution in elution buffer 200 (EB200; 50 mM NaH_2_PO_4_ pH 8, 300 mM NaCl, 200 mM imidazole) and subsequently by a 1.5 mL elution in elution buffer 250 (EB250; 50 mM NaH_2_PO_4_ pH 8, 300 mM NaCl, 250 mM imidazole). Protein overexpression and purity were assessed via SDS-PAGE (4%–20% Mini-PROTEAN^®^ TGX™ Gel, Bio-Rad) analysis and coomassie staining (Figure S25) demonstrating a near homogeneous protein preparation. Eluants containing purified protein were pooled and glycerol was added to 50% (*v*/*v*) for short-term storage at −20 °C.

### 3.11. In Vitro Acetylation of Amicoumacin A

A 1 mL aliquot of purified AmiS protein was buffer exchanged (Amicon Ultra Centrifugal Filter Unit 10 KDa molecular weight cutoff; EMD Millipore, Darmstadt, Germany) into native lysis buffer to remove storage glycerol and was concentrated. This sample was further buffer exchanged and concentrated into the in vitro reaction buffer (50 mM NaH_2_PO_4_ pH 7, 2 mM tris(2-carboxyethyl)phosphine (TCEP; Sigma, St. Louis, MO, USA)). Any protein aggregates that formed during this process were removed via centrifugation at 20,000× *g* for 5 min at 4 °C. Protein concentration was determined via Bradford assay, and a portion of the protein was subsequently diluted to a working concentration of 5.24 μM and the concentration was re-verified. In vitro reactions were prepared in triplicate on a 50 μL scale containing 50 μM NaH_2_PO_4_ pH 7, 2 mM TCEP, 0.3 mM acetyl-CoA (Sigma), 0.1 mM amicoumacin A (when needed), and 3 nM 6 × His-AmiS (when needed). When the amicoumacin A substrate or enzyme were excluded in the control reactions, DMSO or in vitro reaction buffer served as their replacements, respectively. Reaction components were added in the order listed above to the necessary volume of molecular biology grade water. Reactions were incubated at 25.0 °C for 1 h and then held at 12.0 °C (5 h) until being quenched via extraction with 60 μL of ethyl acetate. Reactions were thoroughly mixed with the added ethyl acetate via vortexing, and the phases were separated by centrifugation at 10,000× *g* for 15 s. 50 μL of ethyl acetate was removed and dried under reduced pressure for 30 min on a Genevac HT-4X evaporation system. The residue was resuspended in 20 μL of methanol. A sample of crude extract (MS) or partially purified crude extract (MS^2^, fraction 30, see Section 3.5. Isolation of Amicoumacin Metabolites **1**, **3** and **4**) was utilized as a positive control. 2 μL of sample was injected and analyzed on an Agilent iFunnel 6550 QTOF MS system equipped with a Phenomenex Kinetex C_18_ (100 Å) 5 μm (4.6 mm × 250 mm) column. Liquid chromatography occurred at 25 °C with a solvent flow rate of 0.7 mL/min using a water:acetonitrile (ACN) gradient solvent system containing 0.1% formic acid: 0–30 min, 10%–100% ACN; hold for 5 min, 100% ACN; 0.1 min, 100%–10% ACN; 10 min re-equilibration post-time, 10% ACN. Mass spectra were acquired in the range of 25–1700 *m*/*z* at a scan rate of 1 spectra/s. A Dual Agilent Jet Stream (AJS) ESI source in positive mode was used with parameters set as follows: Drying Gas Temp, 280 °C; Drying Gas Flow, 11 L/min; Nebulizer Pressure, 40 psig; Sheath Gas Temperature, 350 °C; Sheath Gas Flow, 11 L/min; Fragmentor Voltage, 175 V; Skimmer Voltage, 65 V; OCT 1 RF Vpp, 750 V; Capillary Voltage, 4000 V; and Nozzle Voltage, 2000 V. Additionally, tandem MS (MS^2^) analysis was performed using the acquisition parameters described above with the following changes: Nebulizer Pressure, 50 psig; and Fragmentor Voltage, 200 V. Auto MS^2^ data collection was utilized with a preferred ion list containing the masses corresponding to the protonated forms of amicoumacin A and *N*-acetyl-amicoumacin A. The collision energy was set at fixed levels of 20, 30, 40, 50, 75, and 100 V. Precursor ion masses were allowed a 10 ppm mass range and a 0.5 min retention time margin of error. A maximum of 20 precursor ions were analyzed per cycle with precursor ions having a minimum threshold intensity of 200 counts (absolute) or 0.01% (relative). Isotope models were inactivated. The scan speed was varied according to precursor abundance to allow a target of 25000 counts/spectrum and the MS^2^ accumulation time limit was enabled. Data were acquired and analyzed using MassHunter Workstation Data Acquisition (Version B.05.01, Build 5.01.5125.1, Agilent Technologies) and MassHunter Qualitative Analysis (Version B.06.00, Agilent Technologies), respectively. A composite extracted ion chromatogram (a 10 ppm window around the calculated *m*/*z*) for the protonated forms of amicoumacin A and *N*-acetyl-amicoumacin A was generated for each reaction condition and crude material. Comparison of these traces demonstrated the dependence of *N*-acetyl-amicoumacin A formation on 6 × His-AmiS.

### 3.12. Modeling of N-Acetyl-Amicoumacin A Interactions with the T. thermophilus Ribosome

Amicoumacin A was previously reported to interact with the bacterial ribosome at the E site [14]. The reported crystal structure (PDBID 4W2F) was utilized as a base model. One 70S ribosomal complex of the asymmetric crystal unit was imported into PyMOL (Schrödinger, New York, NY, USA). All sidechains and residues with an atom in a 10 Å window about the amicoumacin A ligand were selected for further analysis and saved to a new file. This structure was then imported into Molecular Operating Environment (MOE, 2014.9, Chemical Computing Group, Montreal, QC, Canada). All appropriate residues were protonated utilizing the Protonate3D algorithm built into MOE. This algorithm predicts the 4-amino moiety of amicoumacin A to exist as the protonated, ammonium-like species. The Builder feature was used to append an acetyl group onto the 4-amine and the charge on the nitrogen was set to zero to establish an amide linkage. The *N*-acetyl-amicoumacin A structure alone (all other atoms were set to inert) was then properly parameterized and energy minimized utilizing the MMFF9x force field (R-Field solvation was used under standard parameters with “Cutoff” disabled. “Gradient” was set to 0.00001 and “Rigid Water Molecules” was de-selected). The conformational space of the acetyl group was sampled using the Conformational Search feature. A LowModeMD search was applied with standard parameters (Rejection Limit: 100; Iteration Limit: 10,000; RMS Gradient: 0.005; MM Iteration Limit: 500; RMSD Limit: 0.25; Energy Window: 7; and Conformation Limit: 10,000. Chair Conformations were enforced, and strain energy was separated by stereo class). This search returned three conformers. The backbone amicoumacin atoms of the constructed *N*-acetylamicoumacin A were fixed in position as were atoms of the solvent and surrounding ribosomal pocket, and energy minimization was reperformed to assess the minimal energy conformation of the *N*-acetyl moiety in the presence of the ribosome. Finally, the constructed *N*-acetyl amicoumacin A was minimized in the presence of a fixed ribosomal binding pocket. All structures were imported into PyMOL where polar contacts were predicted (standard cutoff: for ideal geometries 3.6 Å and for minimally acceptable geometries 3.2 Å) and interactions were visualized.

## 4. Conclusions

In summary, we identified the amicoumacin family of antibiotics in the entomopathogen *Xenorhabdus bovienii*. To stimulate metabolite production, we used a bacterial culture medium designed to mimic the amino acid content of insect hemolymph, HMM. Genome synteny and comparative biosynthetic gene cluster analyses revealed that the identified *X. bovienii* amicoumacin gene cluster shares substantial protein homologies among the amicoumacin and xenocoumacin pathways in *B. subtilis* and *X. nematophila*, respectively. There were also some important differences. Specifically, presence of *N*-acetyltransferase AmiS in *X. bovienii* accounts for *N*-acetylation of the amicoumacin scaffold, which was validated through in vitro protein biochemical studies. *N*-acetylation destroyed amicoumacin A’s antibacterial activity, indicating that AmiS serves as a new amicoumacin resistance protein. Ribosomal modeling of *N*-acetyl-amicoumacin A suggests that this *N*-acyl appendage destabilizes interactions with the ribosome, supporting a mechanism for amicoumacin resistance. Larger *N*-acyl modifications, such as a *N*-acyl-d-Asn moiety in the inactive pro-drug scaffold, would present prohibitive steric clashes in the amicoumacin-ribosome structure, supporting a steric ribosomal exclusion mechanism for the preamicoumacins. Indeed, *N*-acyl cleavage and formation appear to be enzymatic strategies for the activation and attenuation of the amicoumacin antibiotics.

## Figures and Tables

**Table 1:** Proteins encoded by the amicoumacin biosynthetic gene cluster in *X. bovienii* str. feltiae Moldova, their proposed functions, and their homology to biosynthetic genes in *X. nematophila* ATCC 19061 and *B. subtilis* subsp. inaquosorum KCTC 13429.

Protein	Size ^a^	Predicted Function	Homolog ^b^	*X. nematophila* (%)		*B. subtilis* (%)	
				Identity	Similarity	Identity	Similarity
AmiA	1488	NRPS	XcnA ^c^	23.3	38.8	30.3	49.2
AmiB	497	Peptidase ^d^	XcnG	55.1	71.8	30.1	46.9
AmiD	243	Thioesterase	XcnI	60.5	74.5	31.3	52.0
AmiE	282	Dehydrogenase ^e^	XcnB	74.8	86.2	49.3	67.8
AmiF	352	Acyl carrier protein ^f^	XcnC	79.8	90.6	53.0	74.0
AmiG	85	Acyl carrier protein	XcnD	76.5	92.9	44.7	73.7
AmiH	382	Dehydrogenase ^g^	XcnE	83.1	93.4	49.0	70.8
AmiI-1	1051	NRPS	XcnA *^c^*	24.5	43.5	33.5	54.1
AmiI-2	1924	PKS	XcnH	72.6	85.2	37.6	55.1
AmiJ	858	NRPS	XcnK	61.9	76.5	30.9	49.6
AmiK	1487	PKS	XcnL	66.3	81.2	36.0	54.7
AmiL-M	3419	PKS	XcnF	68.1	81.8	34.8 *^i^*	50.3 ^i^
AmiP	46	Hypothetical	--	--	--	--	--
AmiQ	461	Drug transporter ^h^	--	--	--	--	--
AmiR	107	Hypothetical	XcnJ	78.5	88.8	--	--
AmiS	152	*N*-acetyltransferase	--	--	--	--	--

^a^ amino acid (aa) number, ^b^ *X. nematophila*, ^c^ partial, ^d^ *N*-acyl-d-asparagine specific peptidase, ^e^ 3-hydroxyacyl-CoA dehydrogenase, ^f^ methoxymalonate biosynthesis protein, ^g^ acyl-CoA dehydrogenase, ^h^ multidrug and toxic compound extrusion (MATE) protein, ^i^ Represented as an average value of AmiL-M to AmiL and AmiM from *B. subtilis*.

## Supplementary Materials

Supplementary materials can be accessed at: http://www.mdpi.com/1420-3049/21/7/824/s1.
