# Supplementary material for: Activating and Attenuating the Amicoumacin Antibiotics
Source: Molecules. 2016 Jun 24;21(7):824. doi: 10.3390/molecules21070824 (PMC5055758; doi:10.3390/molecules21070824)
Supplement: Supplementary file 1 [file molecules-21-00824-s001.pdf]

Supplementary Materials: Activating and Attenuating the Amicoumacin Antibiotics

Hyun Bong Park, Corey E. Perez, Elena Kim Perry and Jason M. Crawford

Table S1. Hemolymph-mimetic media formulation from *Galleria mellonella.*

Amino Acid	3-Letter	Molecular Weight	mM	g/L	g/500 mL
Alanine	Ala	89.09318	29.8	2.65	1.33
Arginine	Arg	174.2	6.24	1.09	0.54
Asparagine	Asn	132.1179	5.71	0.75	0.38
Aspartic acid	Asp	133.11	0.5	0.07	0.03
Cysteine	Cys	121.16	0.41	0.05	0.02
Glutamic acid	Glu	147.13	0	0.00	0.00
Glutamine	Gln	146.14	99.8	14.58	7.29
Glycine	Gly	75.0666	21.2	1.59	0.80
Histidine	His	155.1546	5.98	0.93	0.46
Isoleucine	Ile	131.18	3.72	0.49	0.24
Leucine	Leu	131.18	5.13	0.67	0.34
Lysine	Lys	146.19	6.67	0.98	0.49
Methionine	Met	149.21	0.89	0.13	0.07
Phenylalanine	Phe	165.19	1.64	0.27	0.14
Proline	Pro	115.13	72.6	8.36	4.18
Serine	Ser	105.09	8.07	0.85	0.42
Threonine	Thr	119.1192	4.72	0.56	0.28
Tryptophan	Trp	204.225	0	0.00	0.00
Tyrosine	Tyr	181.19	0.71	0.13	0.06
Valine	Val	117.15	7.33	0.86	0.43
+ Yeast extract				5	2.5

Table S2. BLASTP Analysis of AmiS.

**Sequences Producing Significant Alignments**						
**Description**	**Max Score**	**Total Score**	**Query Cover**	**E value**	**Ident**	**Accession**
GNAT family *N*-acetyltransferase [*Xenorhabdus bovienii*]	315	315	100%	2.00E-109	100%	WP_051863037.1
GNAT family *N*-acetyltransferase [*Xenorhabdus bovienii*]	314	314	100%	6.00E-109	99%	WP_046335894.1
GNAT family *N*-acetyltransferase [*Xenorhabdus bovienii*]	312	312	100%	5.00E-108	99%	WP_051875239.1
GNAT family *N*-acetyltransferase [*Xenorhabdus bovienii*]	288	288	100%	1.00E-98	89%	WP_012988265.1
GNAT family N-acetyltransferase [*Xenorhabdus bovienii*]	288	288	100%	3.00E-98	88%	WP_038257591.1
hypothetical protein KS18_09945 [*Photorhabdus luminescens*]	173	173	86%	3.00E-53	61%	KGM28220.1
GNAT family *N*-acetyltransferase [*Photorhabdus luminescens*]	173	173	86%	4.00E-53	61%	WP_046395321.1
acetyltransferase [*Photorhabdus luminescens* BA1]	173	173	89%	4.00E-53	60%	EYU15535.1
hypothetical protein [*Photorhabdus luminescens*]	172	172	98%	9.00E-53	55%	WP_049584454.1
hypothetical protein TI10_16375 [*Photorhabdus luminescens* subsp. *luminescens*]	170	170	86%	4.00E-52	60%	KMW72247.1
hypothetical protein KS43_19775 [*Pectobacterium carotovorum* subsp. *odoriferum*]	167	167	86%	7.00E-51	53%	KGA31080.1
GNAT family *N*-acetyltransferase [*Pectobacterium carotovorum*]	167	167	88%	8.00E-51	52%	WP_044208192.1
hypothetical protein BCS7_13555 [*Pectobacterium carotovorum* subsp. *odoriferum*]	164	164	86%	1.00E-49	53%	AIU90518.1
GNAT family *N*-acetyltransferase [*Spirochaeta cellobiosiphila*]	142	142	88%	5.00E-41	46%	WP_028972975.1
GCN5 family acetyltransferase [*Aliivibrio fischeri*]	141	141	84%	1.00E-40	52%	WP_063659643.1
GNAT family *N*-acetyltransferase [*Vibrio ordalii*]	141	141	93%	2.00E-40	48%	WP_017049057.1
Orfc641-2 [*Aliivibrio fischeri*]	140	140	84%	3.00E-40	50%	AAO38241.1
GCN5 family acetyltransferase [*Vibrio parahaemolyticus*]	140	140	84%	4.00E-40	50%	WP_025611604.1
GCN5 family acetyltransferase [*Marinobacterium stanieri*]	140	140	90%	4.00E-40	50%	WP_010322146.1
GCN5 family acetyltransferase [*Vibrio furnissii*]	139	139	86%	2.00E-39	48%	WP_038152049.1
GCN5 family acetyltransferase [*Vibrio furnissii*]	138	138	86%	2.00E-39	47%	WP_041943101.1
GCN5 family acetyltransferase [*Halomonas halocynthiae*]	138	138	88%	3.00E-39	47%	WP_027967474.1
GCN5 family acetyltransferase [*Vibrio alginolyticus*]	138	138	86%	3.00E-39	48%	WP_053308726.1
GCN5 family acetyltransferase [*Aliivibrio fischeri*]	137	137	84%	4.00E-39	50%	WP_005422878.1
GCN5 family acetyltransferase [*Vibrio* sp. RC341]	137	137	86%	5.00E-39	47%	WP_000619633.1
hypothetical protein [*Pseudomonas* sp. TTU2014-080ASC]	137	137	88%	6.00E-39	47%	WP_058068371.1
GCN5 family acetyltransferase [*Vibrio natriegens*]	137	137	87%	8.00E-39	47%	WP_020333123.1
GCN5 family acetyltransferase [*Vibrio nigripulchritudo*]	137	137	85%	8.00E-39	47%	WP_004403585.1
GCN5 family acetyltransferase [*Vibrio parahaemolyticus*]	137	137	86%	8.00E-39	47%	WP_025634936.1
GCN5 family acetyltransferase [*Vibrio cholerae*]	137	137	86%	8.00E-39	47%	WP_000620002.1
acetyltransferase [*Vibrio nigripulchritudo*]	136	136	85%	9.00E-39	47%	WP_022560845.1
GCN5 family acetyltransferase [*Vibrio cholerae*]	136	136	86%	1.00E-38	48%	WP_042988829.1
GCN5 family acetyltransferase [*Vibrio cholerae*]	136	136	86%	1.00E-38	47%	WP_057563832.1

GCN5 family acetyltransferase [*Vibrio parahaemolyticus*]	136	136	84%	1.00E-38	48%	WP_031821635.1
MULTISPECIES: GCN5 family acetyltransferase [*Vibrio*]	136	136	86%	1.00E-38	47%	WP_000620001.1
acetyltransferase [*Vibrio nigripulchritudo*]	136	136	85%	1.00E-38	47%	WP_022612737.1
GCN5 family acetyltransferase [*Vibrio jasicida*]	136	136	86%	1.00E-38	47%	WP_038882262.1
GCN5 family acetyltransferase [*Vibrio orientalis*]	136	136	86%	1.00E-38	47%	WP_004417641.1
acetyltransferase [*Vibrio nigripulchritudo*]	136	136	85%	1.00E-38	47%	WP_022590887.1
GCN5 family acetyltransferase [*Vibrio sinaloensis*]	136	136	84%	1.00E-38	49%	WP_039485629.1
MULTISPECIES: GCN5 family acetyltransferase [*Vibrio*]	135	135	84%	2.00E-38	49%	WP_038939720.1
GCN5 family acetyltransferase [*Vibrio parahaemolyticus*]	135	135	86%	2.00E-38	47%	WP_045604836.1
GCN5 family acetyltransferase [*Vibrio parahaemolyticus*]	135	135	84%	2.00E-38	47%	WP_029817191.1
GCN5 family acetyltransferase [*Vibrio* sp. EJY3]	135	135	88%	2.00E-38	46%	WP_014231659.1
GCN5 family acetyltransferase [*Vibrio diazotrophicus*]	135	135	96%	2.00E-38	46%	WP_042482237.1
GCN5 family acetyltransferase [*Vibrio parahaemolyticus*]	135	135	86%	3.00E-38	47%	WP_025590712.1
GCN5 family acetyltransferase [*Vibrio parahaemolyticus*]	135	135	84%	3.00E-38	48%	WP_025639322.1
GCN5 family acetyltransferase [*Vibrio* genomosp. F10]	135	135	84%	3.00E-38	48%	WP_017037397.1
GCN5 family acetyltransferase [*Vibrio parahaemolyticus*]	135	135	86%	4.00E-38	47%	WP_053340085.1
GCN5 family acetyltransferase [*Vibrio parahaemolyticus*]	135	135	96%	4.00E-38	44%	WP_025543545.1
GCN5 family acetyltransferase [*Vibrio parahaemolyticus*]	135	135	84%	4.00E-38	49%	WP_029807736.1
MULTISPECIES: GCN5 family acetyltransferase [*Gammaproteobacteria*]	135	135	84%	4.00E-38	48%	WP_006083857.1
GCN5 family acetyltransferase [*Vibrio parahaemolyticus*]	135	135	84%	5.00E-38	48%	WP_025527007.1
GCN5 family acetyltransferase [*Vibrio parahaemolyticus*]	135	135	86%	5.00E-38	47%	WP_042764686.1
GCN5 family acetyltransferase [*Vibrio furnissii*]	135	135	86%	5.00E-38	47%	WP_055466951.1
GCN5 family acetyltransferase [*Vibrio* sp. ZOR0018]	135	135	86%	5.00E-38	47%	WP_047691187.1
GCN5 family acetyltransferase [*Vibrio parahaemolyticus*]	134	134	86%	5.00E-38	47%	WP_029853409.1
GCN5 family acetyltransferase [*Vibrio parahaemolyticus*]	134	134	86%	6.00E-38	47%	WP_053807631.1
GCN5 family acetyltransferase [*Vibrio cholerae*]	134	134	86%	6.00E-38	47%	WP_000592976.1
GCN5 family acetyltransferase [*Vibrio parahaemolyticus*]	134	134	84%	9.00E-38	47%	WP_031410933.1
GCN5 family acetyltransferase [*Vibrio parahaemolyticus*]	134	134	86%	1.00E-37	47%	WP_029804434.1
GCN5 family acetyltransferase [*Vibrio vulnificus*]	134	134	84%	1.00E-37	47%	WP_011080310.1
MULTISPECIES: GNAT family acetyltransferase [*Idiomarina*]	134	134	98%	1.00E-37	42%	WP_058576320.1
GNAT family *N*-acetyltransferase [*Halomonas stevensii*]	134	134	98%	1.00E-37	43%	WP_016914635.1
GCN5 family acetyltransferase [*Vibrio* genomosp. F10]	134	134	84%	1.00E-37	48%	WP_017039718.1
GCN5 family acetyltransferase [*Vibrio parahaemolyticus*]	134	134	84%	1.00E-37	48%	WP_025502523.1
probable acetyltransferase [*Vibrio orientalis* CIP 102891 = ATCC 33934]	133	133	82%	1.00E-37	48%	EEX92052.1
GCN5 family acetyltransferase [*Vibrio parahaemolyticus*]	134	134	86%	2.00E-37	46%	WP_024701852.1
GCN5 family acetyltransferase [*Vibrio parahaemolyticus*]	133	133	86%	2.00E-37	46%	WP_031776680.1
MULTISPECIES: GCN5 family acetyltransferase [*Vibrio*]	133	133	84%	2.00E-37	47%	WP_017048740.1
GCN5 family acetyltransferase [*Vibrio nereis*]	133	133	84%	2.00E-37	47%	WP_053397255.1

GNAT family acetyltransferase [*Thioalkalivibrio sulfidiphilus*]	133	133	86%	2.00E-37	45%	WP_012639157.1
GCN5 family acetyltransferase [*Vibrio parahaemolyticus*]	133	133	84%	2.00E-37	48%	WP_053302539.1
GCN5-related *N*-acetyltransferase [*Spirochaeta smaragdinae* DSM 11293]	133	133	94%	2.00E-37	44%	ADK83259.1
acetyltransferase [*Vibrio proteolyticus*]	133	133	86%	3.00E-37	46%	WP_021704954.1
MULTISPECIES: GNAT family acetyltransferase [*Pseudomonas*]	133	133	92%	3.00E-37	43%	WP_026088471.1
GNAT family acetyltransferase [*Pseudomonas pseudoalcaligenes*]	132	132	88%	3.00E-37	43%	KJU79693.1
GCN5 family acetyltransferase [*Shewanella baltica*]	132	132	84%	3.00E-37	47%	WP_012587276.1
GNAT family *N*-acetyltransferase [*Serpens flexibilis*]	132	132	87%	4.00E-37	42%	WP_039561772.1
GNAT family *N*-acetyltransferase [*Pseudomonas tuomuerensis*]	132	132	87%	5.00E-37	42%	WP_039606303.1
GNAT family *N*-acetyltransferase [*Zooshikella ganghwensis*]	132	132	88%	5.00E-37	44%	WP_027710230.1
GCN5 family acetyltransferase [*Vibrio parahaemolyticus*]	132	132	86%	6.00E-37	47%	WP_025613242.1
GCN5 family acetyltransferase [*Vibrio parahaemolyticus*]	132	132	86%	6.00E-37	46%	WP_025523842.1
acetyltransferase family protein [*Vibrio cholerae* HC-55A1]	132	132	82%	6.00E-37	48%	EKG57792.1
GCN5 family acetyltransferase [*Vibrio rhizosphaerae*]	132	132	86%	7.00E-37	47%	WP_038179468.1
GNAT family acetyltransferase [*Pseudomonas stutzeri*]	132	132	91%	7.00E-37	43%	WP_038658324.1
GCN5 family acetyltransferase [*Vibrio vulnificus*]	132	132	84%	7.00E-37	47%	WP_058645502.1
GCN5 family acetyltransferase [*Vibrio parahaemolyticus* 901128]	131	131	82%	7.00E-37	48%	KIT52952.1
GCN5 family acetyltransferase [*Vibrio xuii*]	132	132	86%	7.00E-37	46%	WP_053441905.1
GNAT family *N*-acetyltransferase [*Aliivibrio logei*]	131	131	86%	1.00E-36	45%	WP_017020570.1
GCN5 family acetyltransferase [*Vibrio parahaemolyticus*]	131	131	84%	1.00E-36	47%	WP_025630340.1
acetyltransferase family protein [*Vibrio parahaemolyticus* VP2007-095]	131	131	82%	1.00E-36	47%	EQM04249.1
GNAT family acetyltransferase [*Serratia rubidaea*]	131	131	86%	1.00E-36	42%	WP_061325446.1
GNAT family acetyltransferase [*Pseudomonas mendocina* EGD-AQ5]	131	131	94%	1.00E-36	42%	ERH47576.1
GCN5 family acetyltransferase [*Vibrio parahaemolyticus*]	131	131	86%	2.00E-36	46%	WP_029858138.1
GCN5 family acetyltransferase [*Vibrio orientalis*]	130	130	86%	2.00E-36	47%	WP_038211432.1
GCN5 family acetyltransferase [*Vibrio cholerae*]	130	130	86%	3.00E-36	46%	WP_032469470.1
GCN5 family acetyltransferase [*Vibrio parahaemolyticus*]	130	130	86%	3.00E-36	46%	WP_029826613.1
GCN5 family acetyltransferase [*Vibrio parahaemolyticus*]	130	130	84%	3.00E-36	47%	WP_025523582.1
GNAT family acetyltransferase [*Spirochaeta smaragdinae*]	130	130	88%	4.00E-36	45%	WP_041866453.1

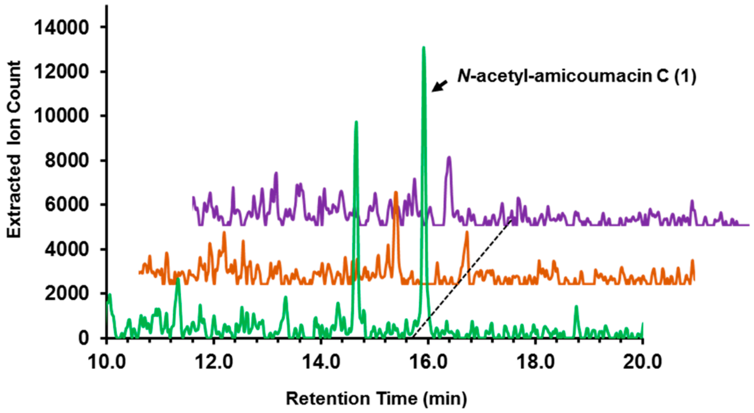

Figure S1. LC/ESI-MS traces of hemolymph-mimetic media (green), LB supplemented with L-proline (brown), and LB (purple). Extracted ion chromatogram was extracted at *m*/*z* 449 $[M + H]^+$ corresponding to *N*-acetyl-amicoumacin C (**1**) ion signal.

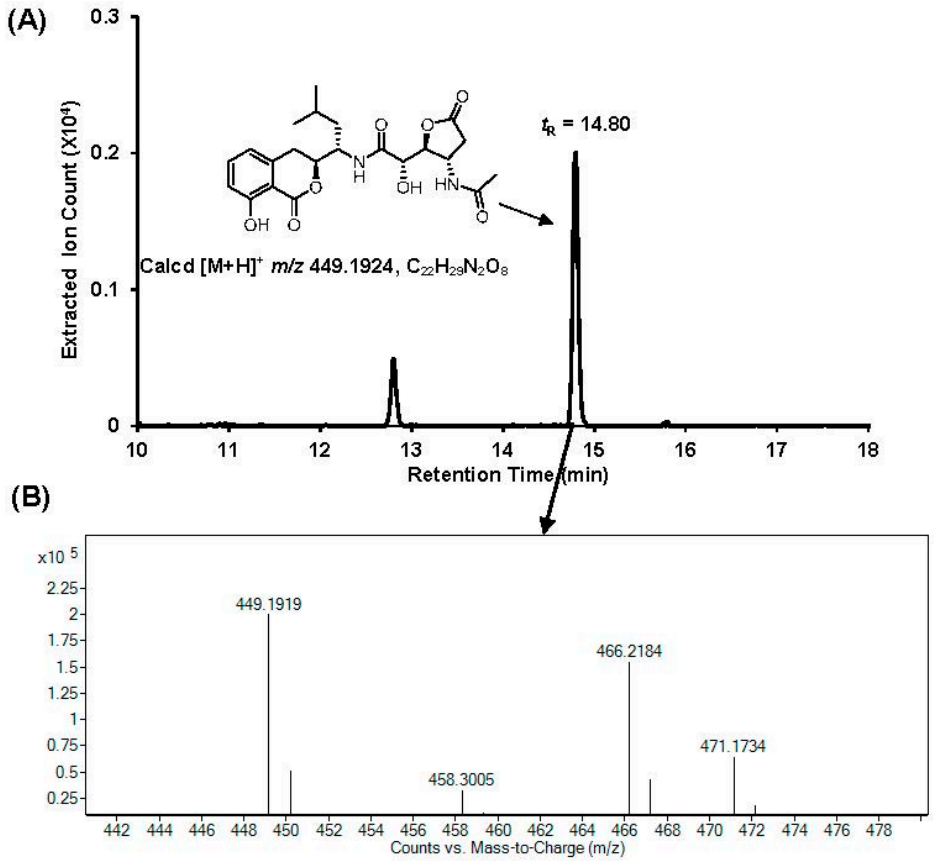

Figure S2. Extracted ion chromatogram (**A**) and HR-ESI-QTOF-MS (**B**) of **1**.

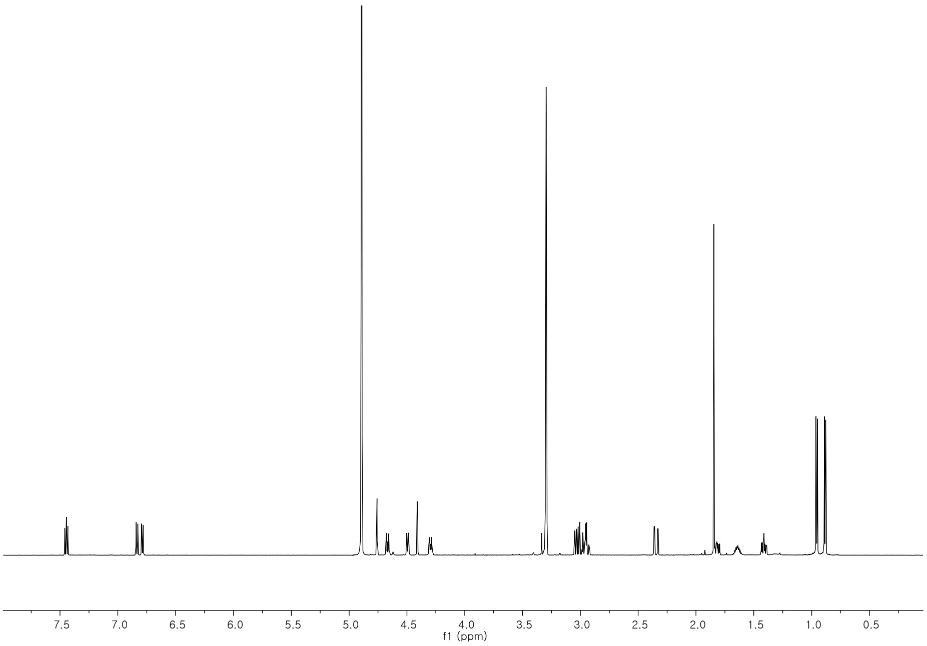

Figure S3. ^{1}H-NMR spectrum of *N*-acetyl-amicoumacin C (**1**) in methanol-*d*4.

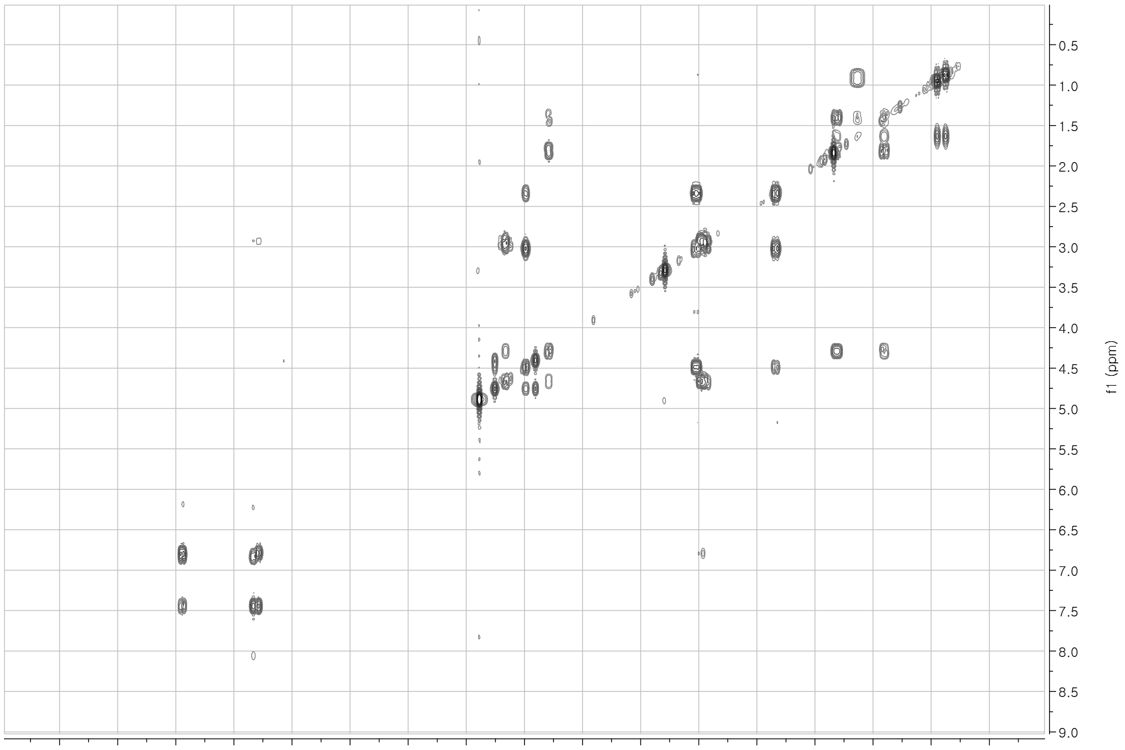

Figure S4. gCOSY NMR spectrum of *N*-acetylamicoumacin C (**1**) in methanol-*d*4.

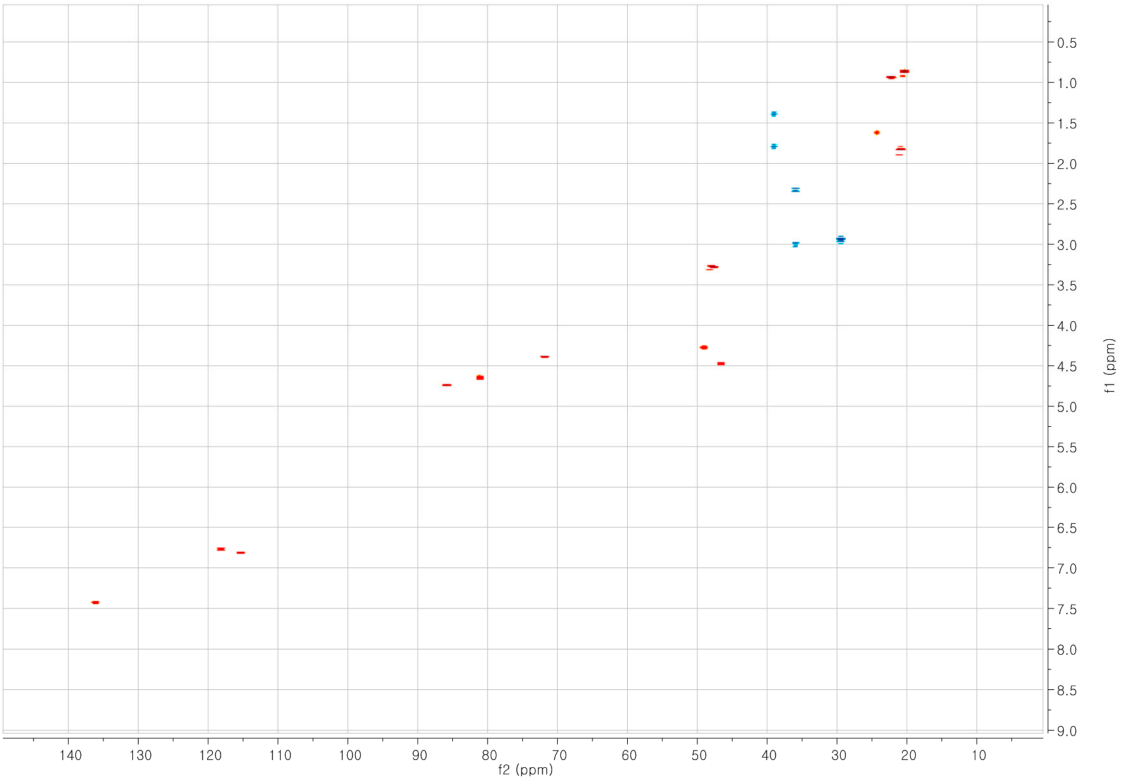

Figure S5. gHSQC NMR spectrum of *N*-acetylamicoumacin C (**1**) in methanol-d_4.

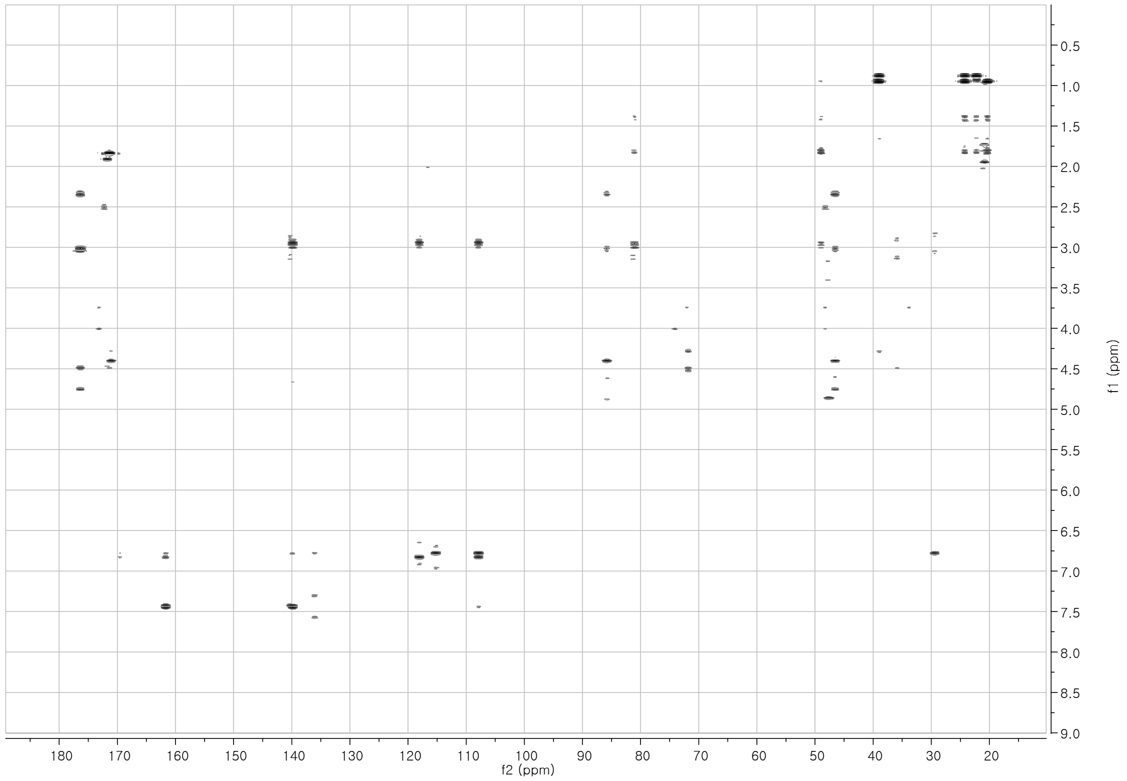

Figure S6. gHMBC NMR spectrum of *N*-acetylamicoumacin C (**1**) in methanol-d_4.

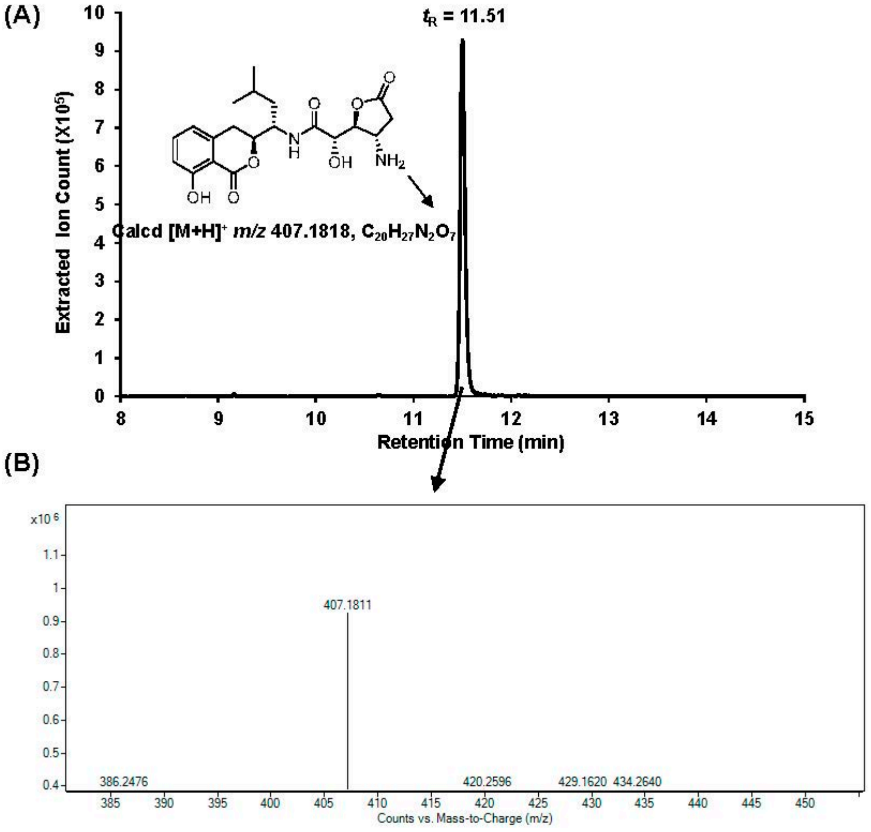

Figure S7. Extracted ion chromatogram (**A**) and HR-ESI-QTOF-MS (**B**) of **2**.

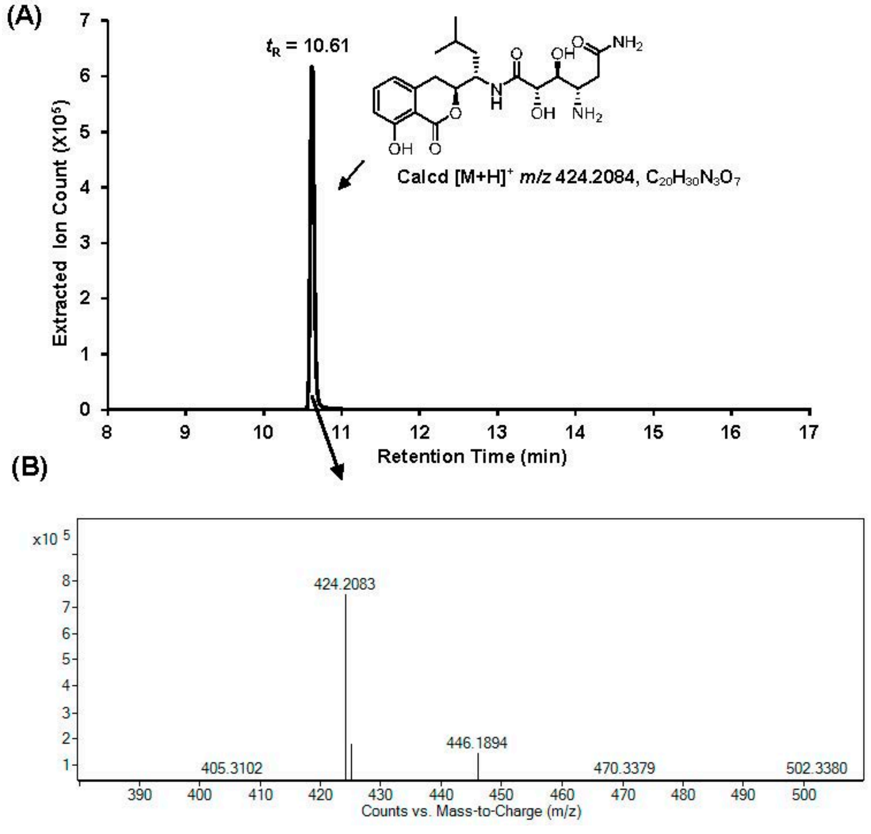

Figure S8. Extracted ion chromatogram (**A**) and HR-ESI-QTOF-MS (**B**) of **4**.

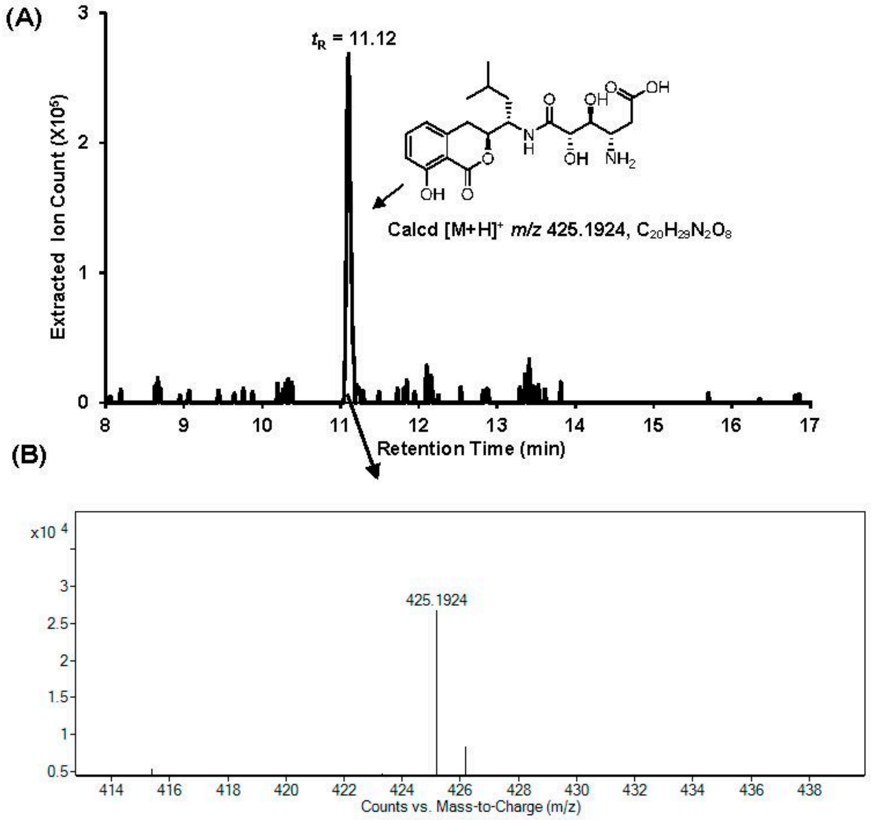

Figure S9. Extracted ion chromatogram (**A**) and HR-ESI-QTOF-MS (**B**) of **6**.

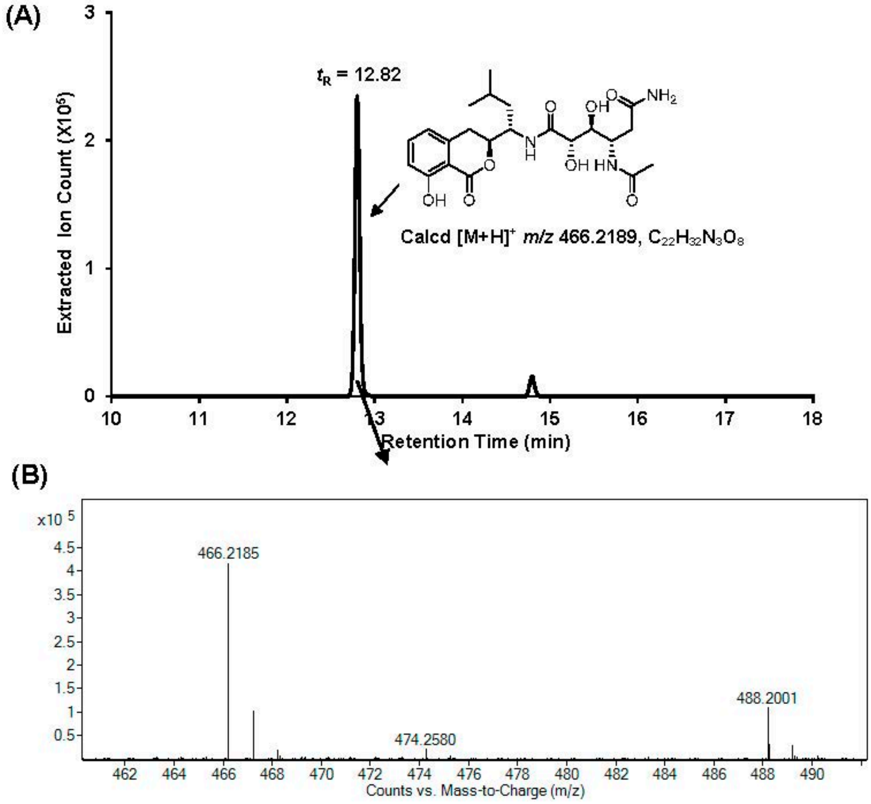

Figure S10. Extracted ion chromatogram (**A**) and HR-ESI-QTOF-MS (**B**) of **3**.

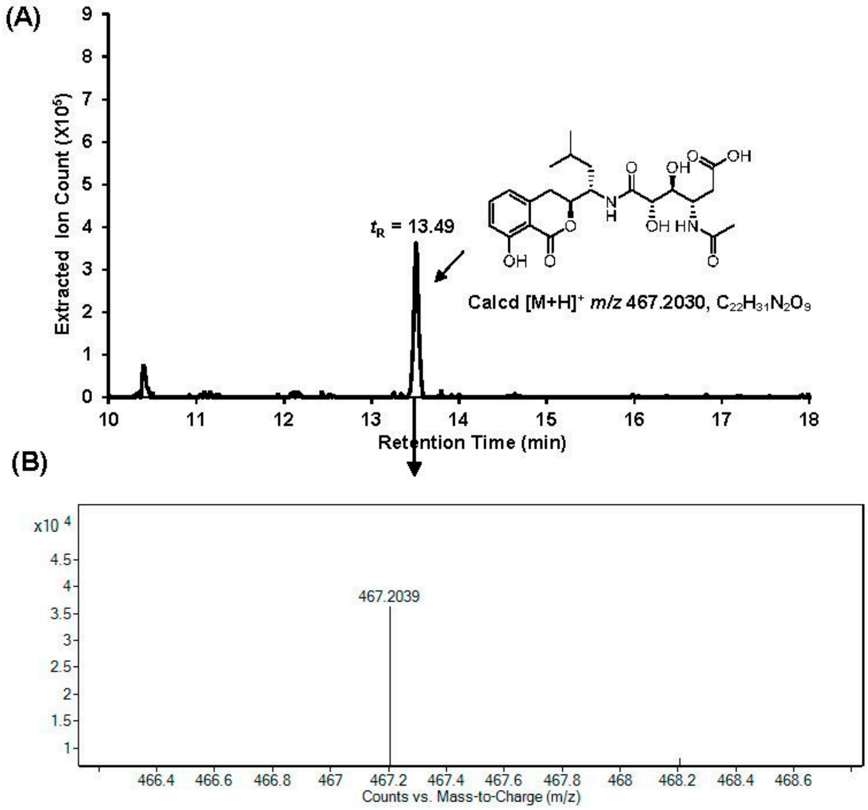

Figure S11. Extracted ion chromatogram (A) and HR-ESI-QTOF-MS (B) of **5**.

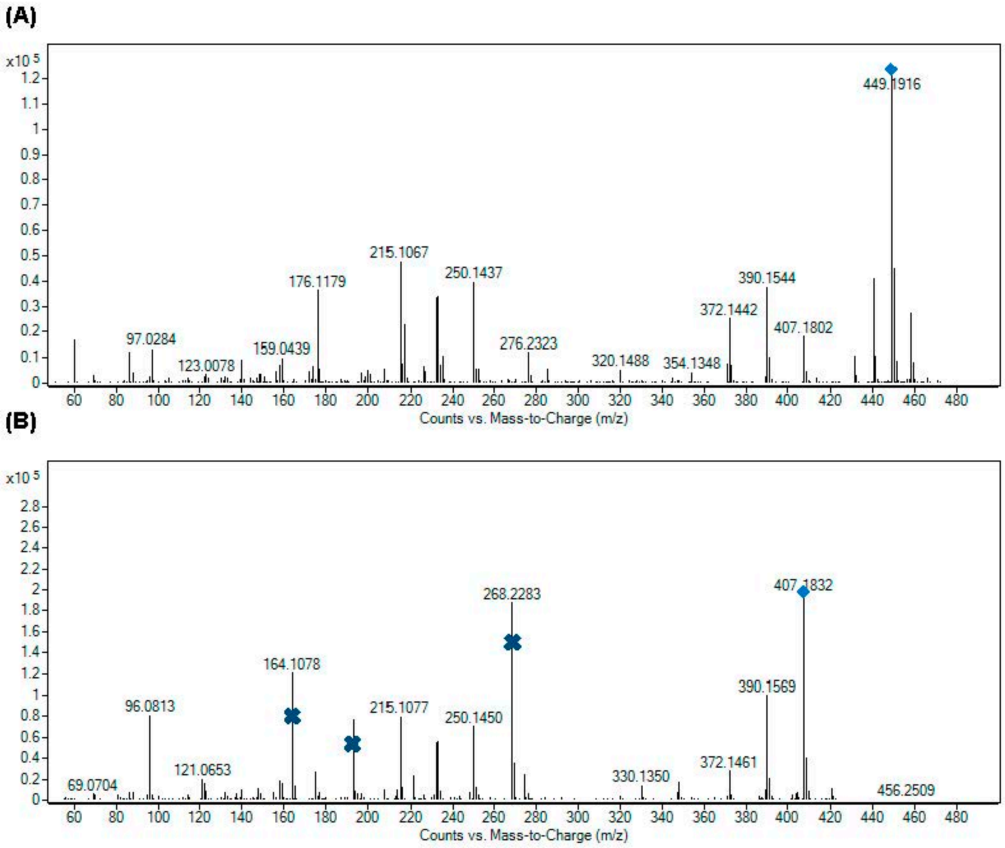

Figure S12. MS² analysis of compounds **1** (**A**) and **2** (**B**). X mark shown in (**B**) indicates impure ion peaks derived from crude extracts.

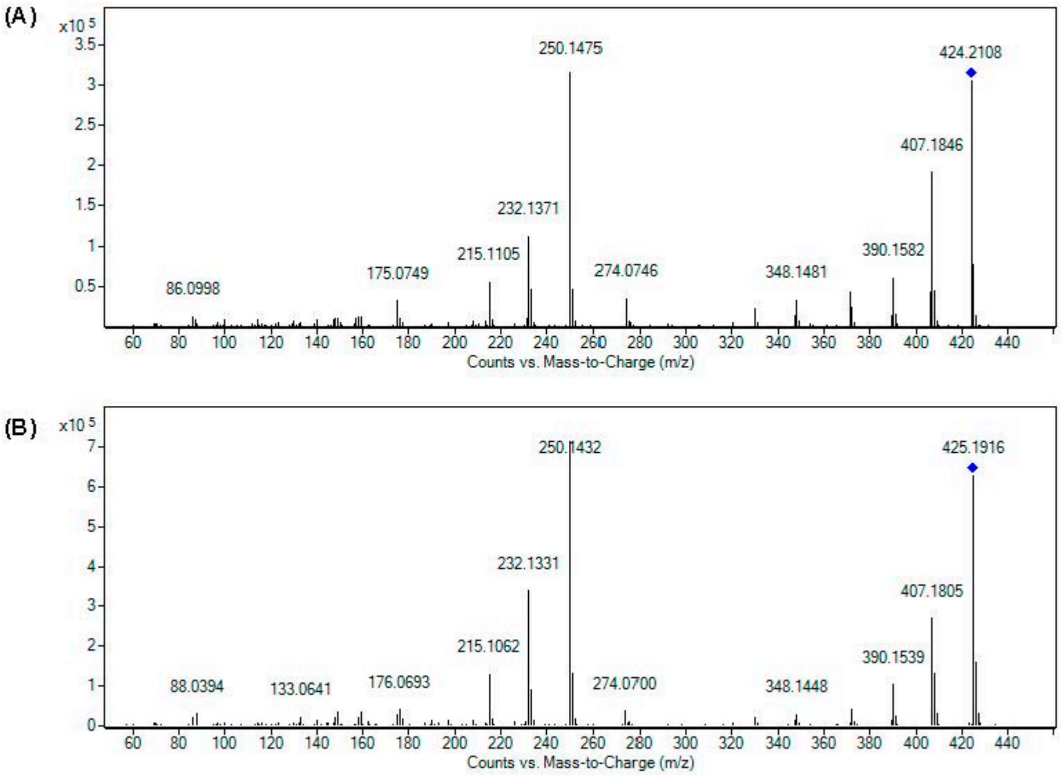

Figure S13. MS^2 analysis of compounds **4** (**A**) and **6** (**B**).

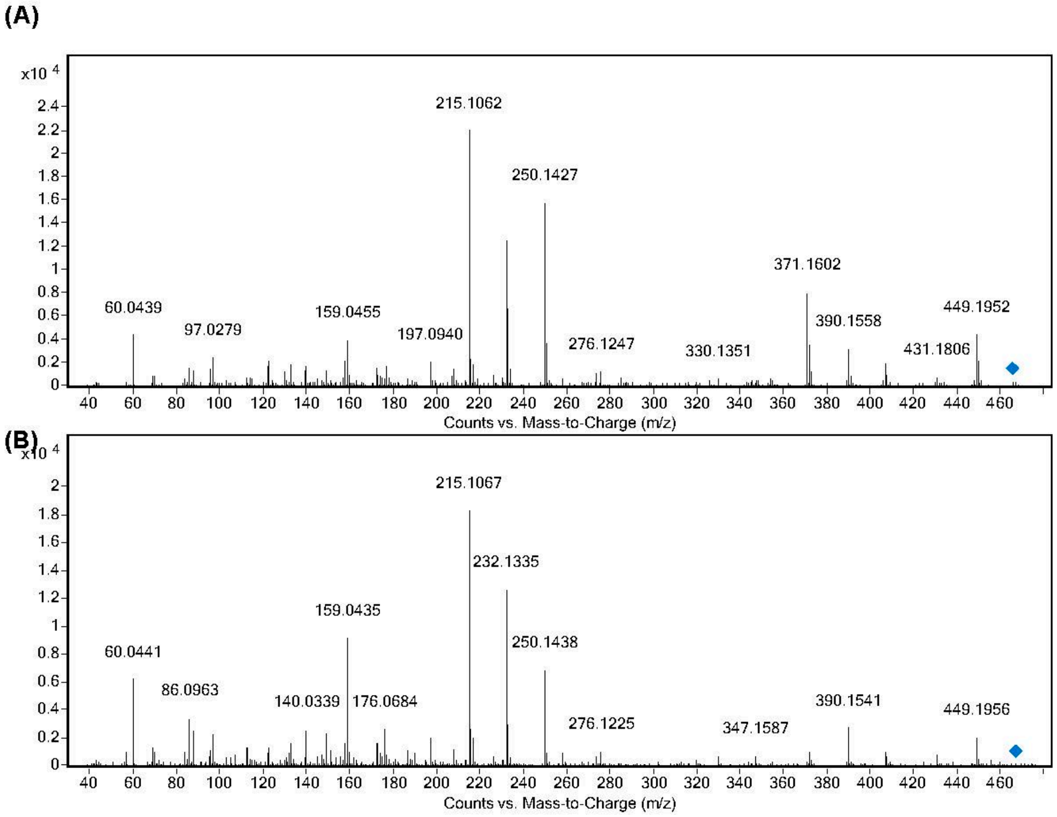

Figure S14. MS^2 analysis of compounds **3** (**A**) and **5** (**B**).

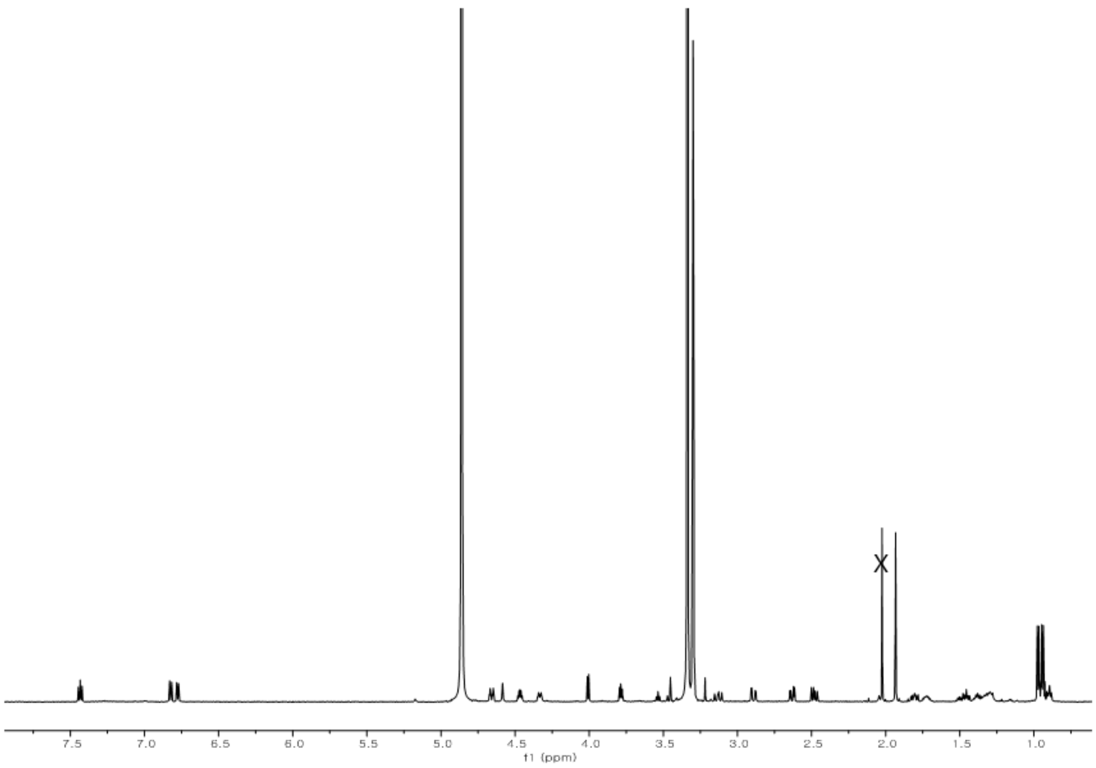

Figure S15. ^{1}H-NMR spectrum of *N*-acetylamicoumacin A (**3**) in methanol-*d*$_4$.

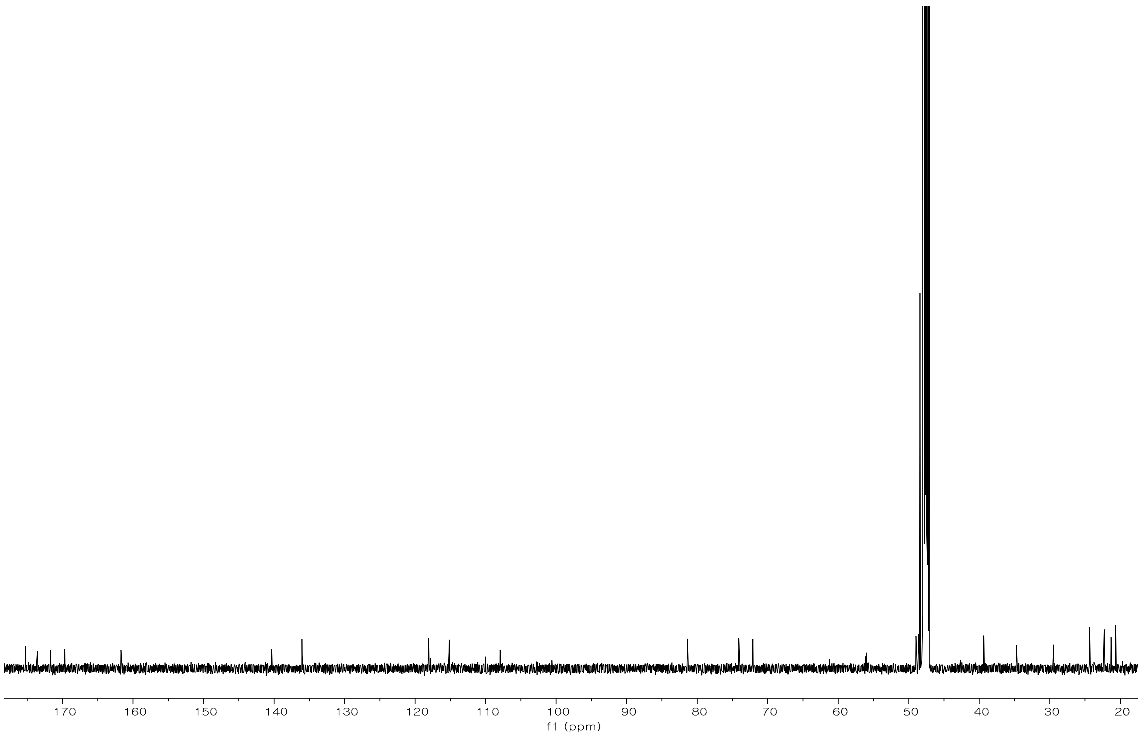

Figure S16. ^{13}C-NMR spectrum of *N*-acetylamicoumacin A (**3**) in methanol-*d*$_4$.

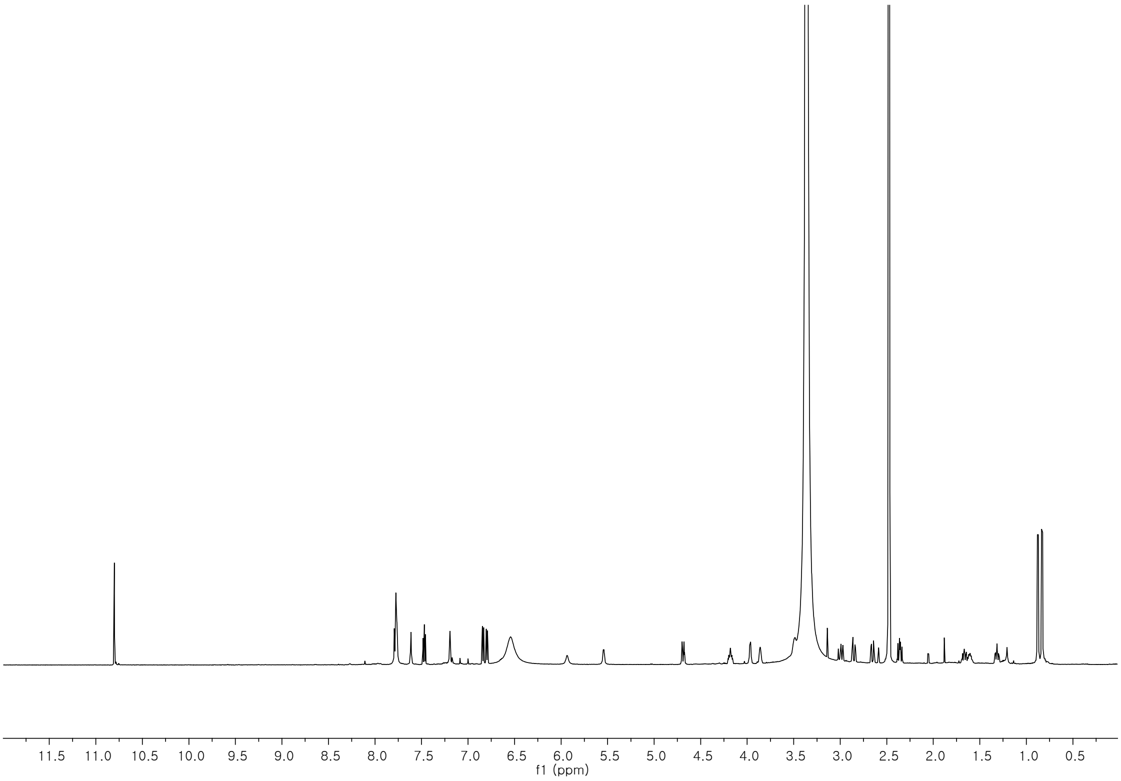

Figure S17. ^{1}H-NMR spectrum of amicoumacin A (**4**) in DMSO-d_6.

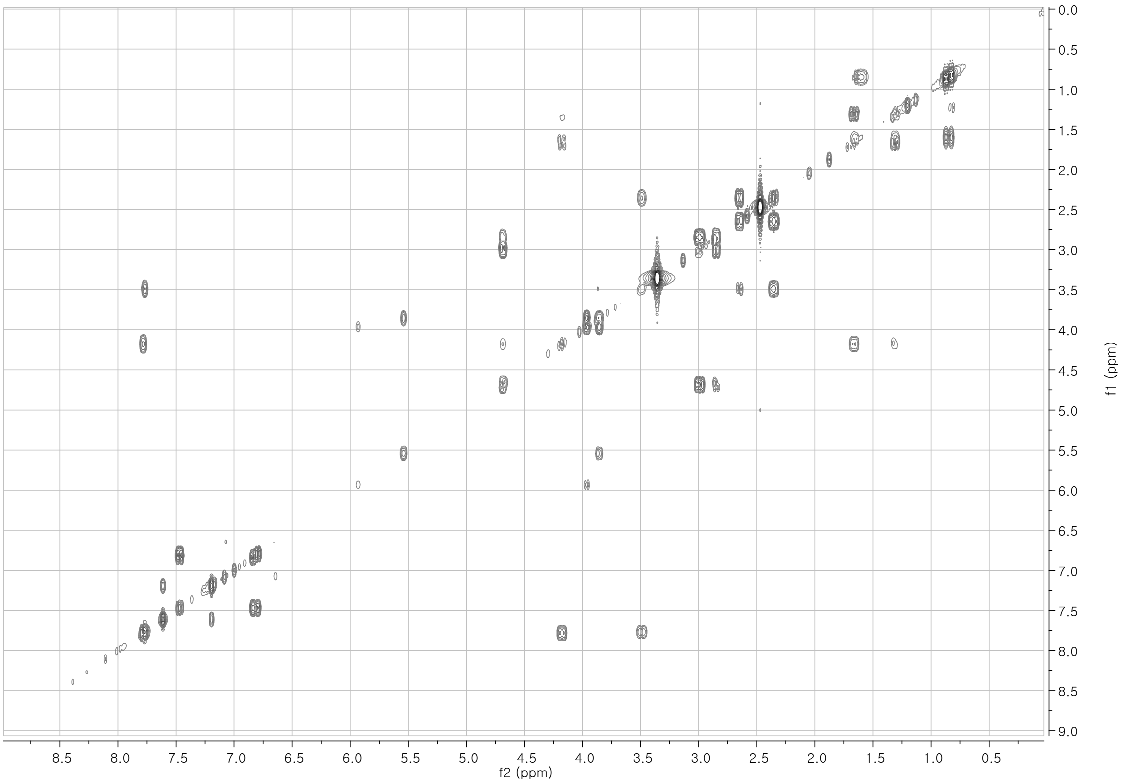

Figure S18. gCOSY NMR spectrum of amicoumacin A (**4**) in DMSO-d_6.

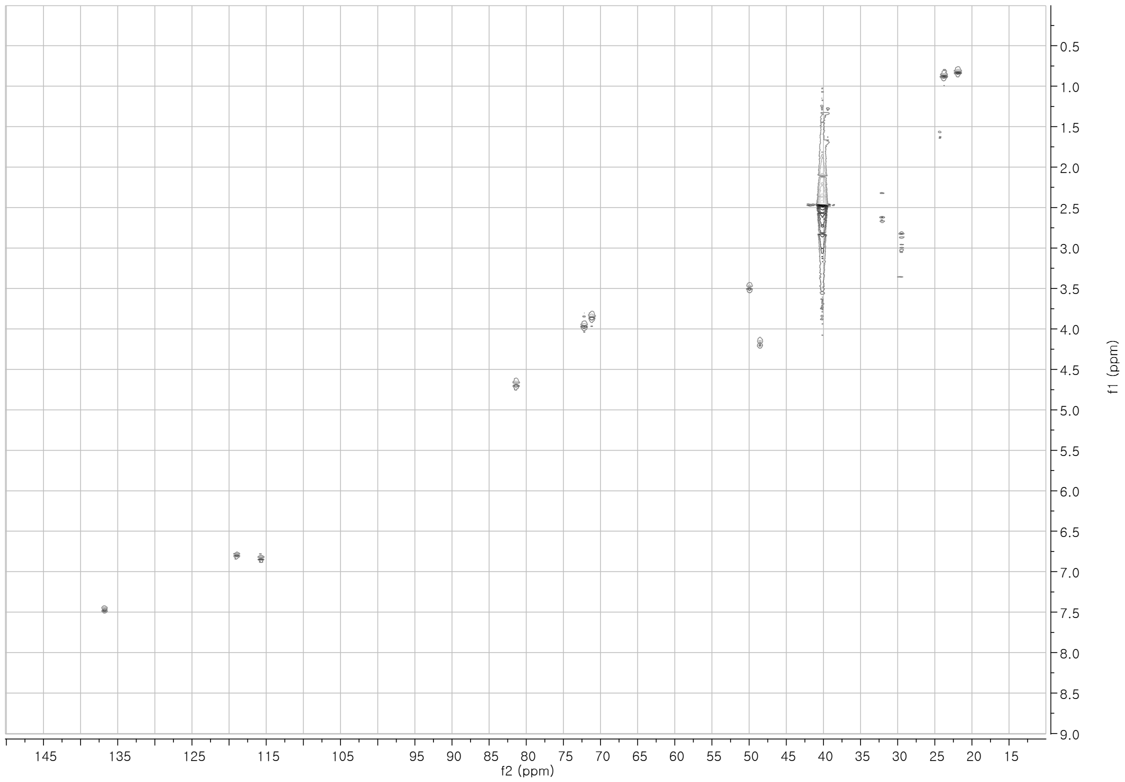

Figure S19. gHSQC NMR spectrum of amicoumacin. A (**4**) in DMSO-*d*6.

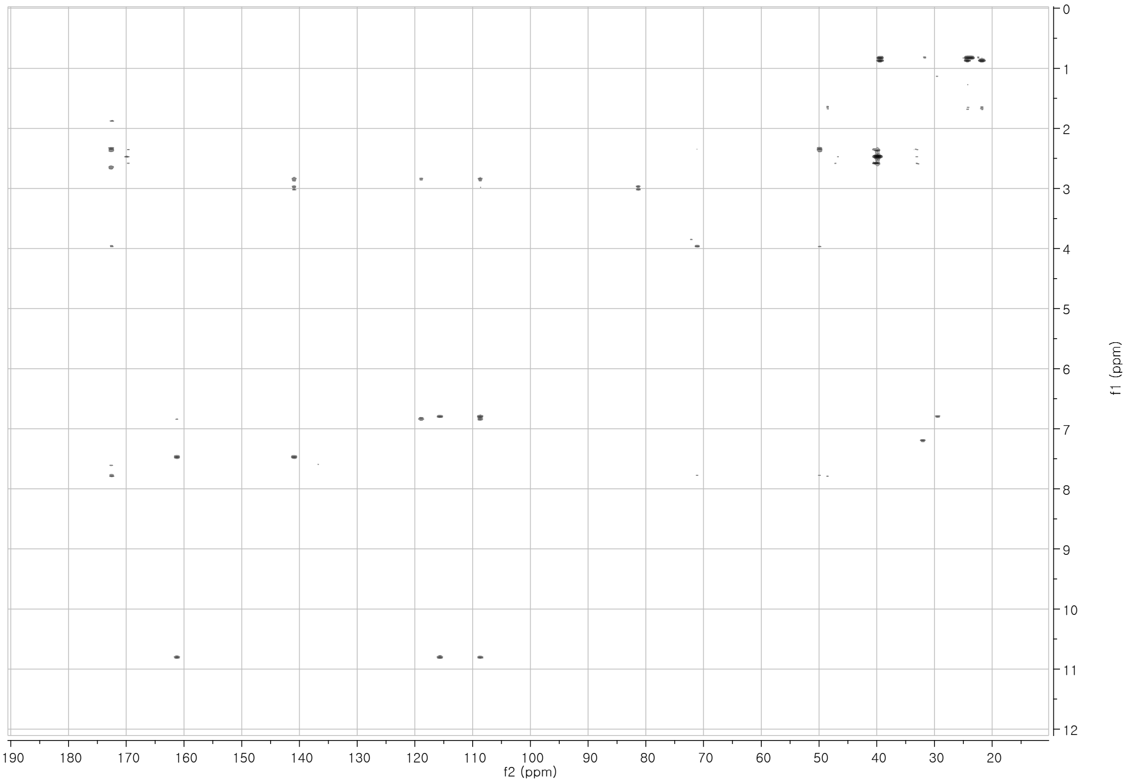

Figure S20. gHMBC NMR spectrum of amicoumacin A (**4**) in DMSO-*d*6.

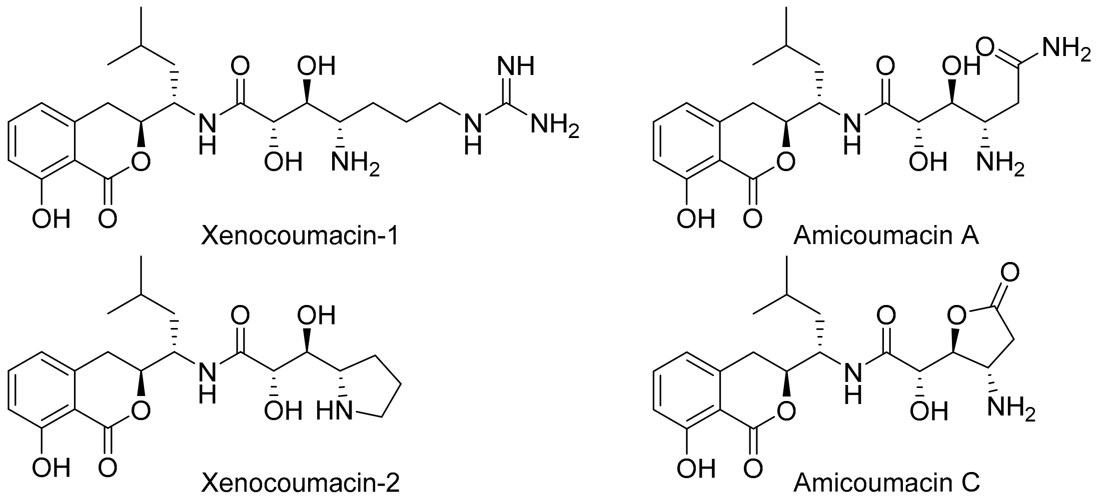

Figure S21. Representative chemical structures of amicoumacins and xenocoumacins.

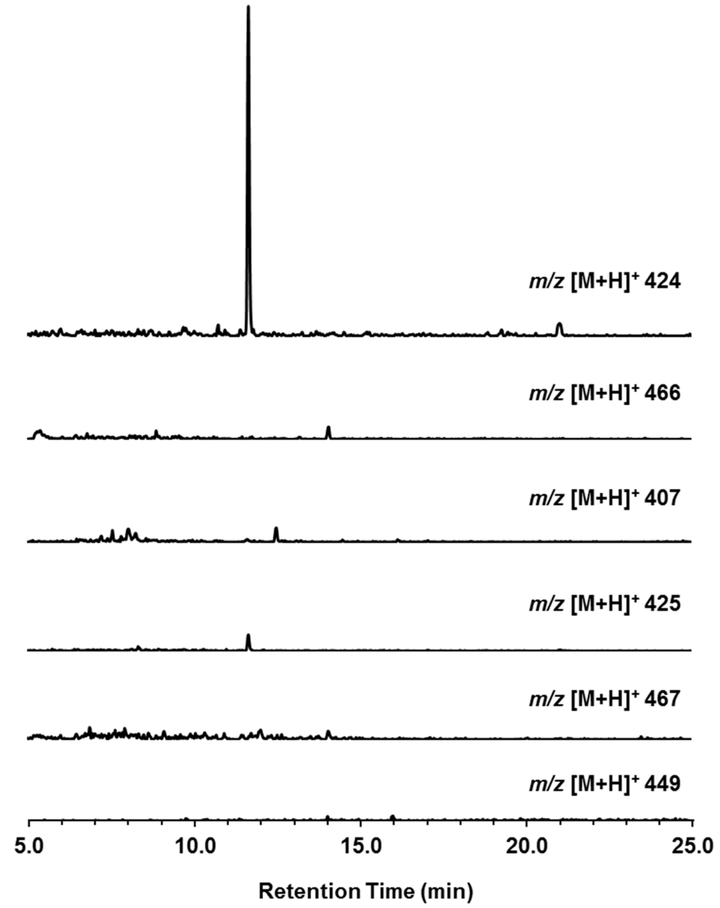

Figure S22. Extracted ion chromatograms of amicoumacin metabolites from XAD-7 extracts.

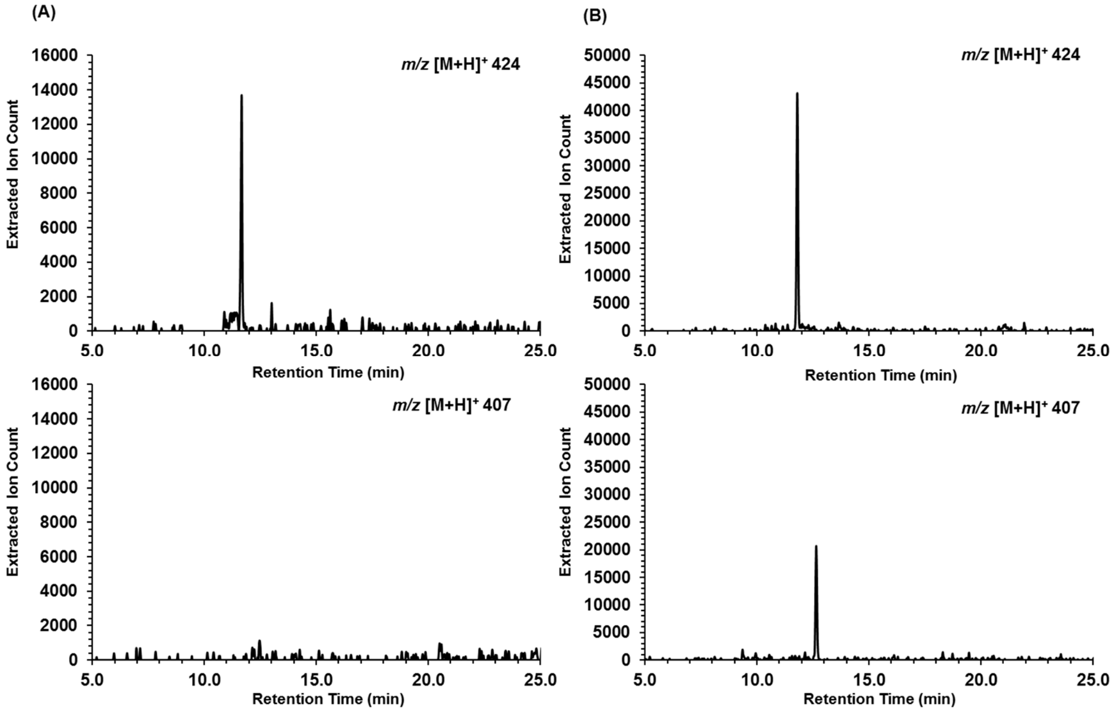

Figure S23. Conversion of amicoumacin A (**4**) into amicoumacin C (**2**) in pure sterilized water (**A**) and cell-free fresh LB liquid medium (**B**).

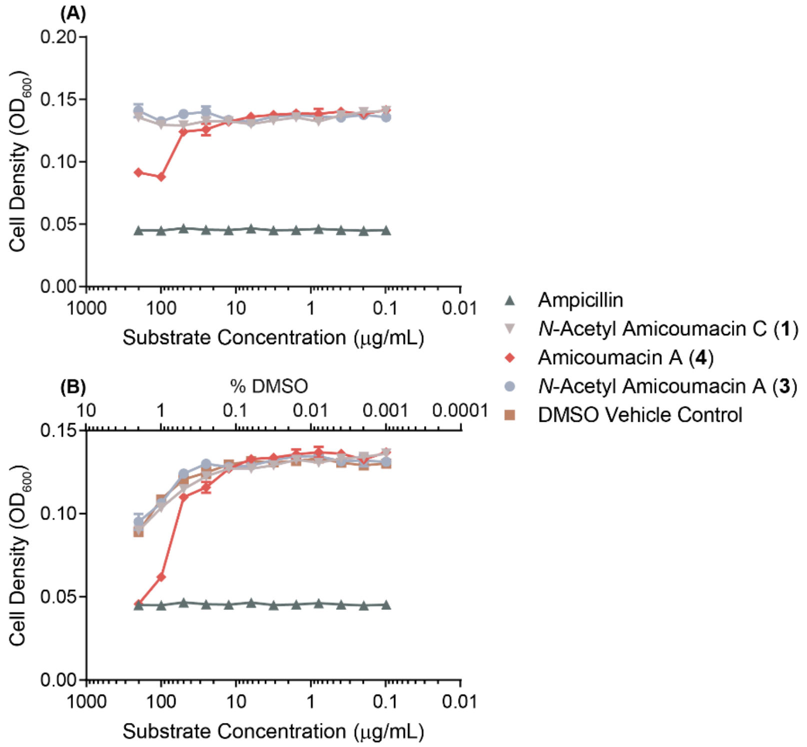

Figure S24. Minimal inhibitory concentration (MIC) analysis of compounds **1**, **3**, **4**. *B. subtilis* was used as the indicator strain. As the vehicle control, DMSO demonstrated growth inhibitory properties at high concentrations (B, upper X-axis). Its contribution to growth inhibition was removed by calculation of this deficit at each concentration relative to the average maximal growth of *B. subtilis* in LB alone (0.135 ± 0.002, n = 12), and addition of this value to the measured cell density of **1**, **3**, and **4** (A). No cell growth was observed in the presence of ampicillin at all tested concentrations (OD_{600} LB Media = 0.046 ± 0.001, n = 12).

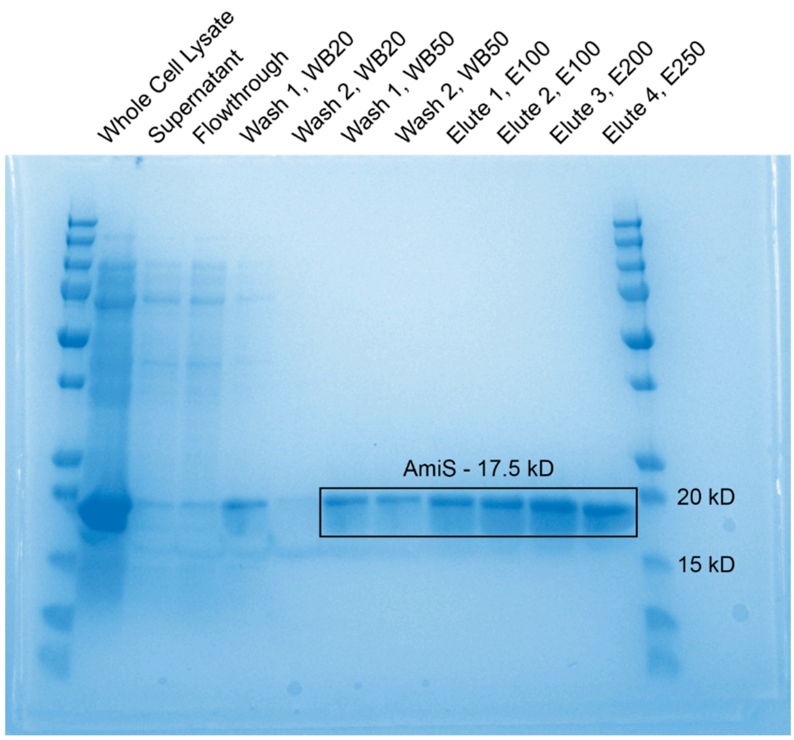

Figure S25. SDS-PAGE analysis of fractions from the purification of 6×His-AmiS.

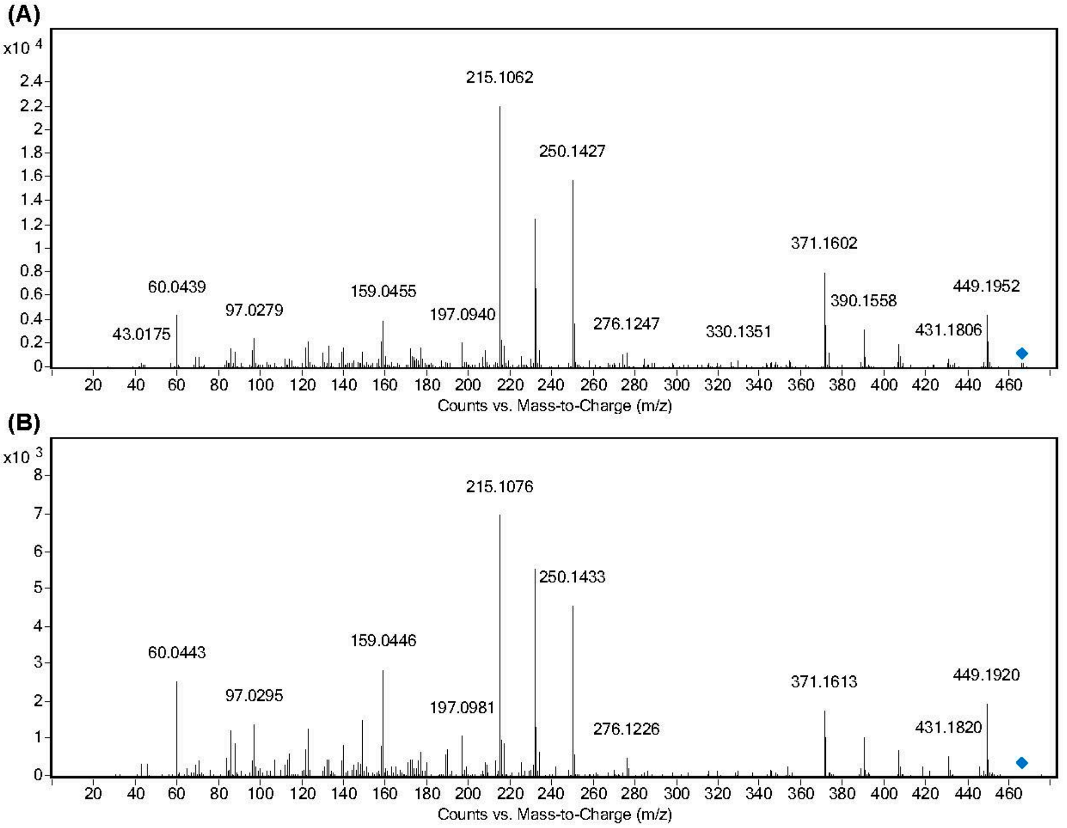

Figure S26. MS[2] analysis (CE = 30 V) of *N*-acetylamicoumacin A (**3**) from partially purified crude extract (**A**) and *in vitro* biosynthesis (**B**). The precursor ion is denoted by the blue diamond.
